# Crystal structure of a binuclear mixed-valence ytterbium complex containing a 2-anthracene-substituted phenoxide ligand

**DOI:** 10.1107/S205698901901154X

**Published:** 2019-08-23

**Authors:** David J. Berg, Brendan Twamley

**Affiliations:** a Department of Chemistry, University of Victoria, PO Box 1700 Stn CSC, Victoria, BC, V8W 2Y2, Canada; b School of Chemistry, Trinity College Dublin, University of Dublin, Dublin 2, Ireland

**Keywords:** crystal structure, aryl­oxide, ytterbium, mixed valence, arene coordination

## Abstract

Reaction of 2-(anthracen-9-yl)phenol (HOPhAn, **1**) with divalent Yb[N(SiMe_3_)_2_]_2_·2THF in THF–toluene mixtures affords the mixed-valence Yb^II^–Yb^III^ dimer {[2-(anthracen-9-yl)phenolato-κ*O*]bis­(tetra­hydro­furan)­ytterbium(III)}-tris­[μ-2-(anthracen-9-yl)phenolato]-κ^4^ *O*:*O*;κ*O*:1,2-η,κ*O*-{[2-(anthracen-9-yl)phenolato-κ*O*]ytterbium(II)} toluene tris­olvate, [Yb_2_(C_20_H_13_O)_5_(C_4_H_8_O)_2_]·3C_7_H_7_ or [Yb^III^(THF)_2_(OPhAn)](μ-OPhAn)_3_[Yb^II^(OPhAn)]·3C_7_H_7_ (**2**), as the major product. It crystallized as a toluene tris­olvate. The Yb—O bond lengths in the crystal structure of this dimer clearly identify the Yb^II^ and Yb^III^ centres. Inter­estingly, the formally four-coordinate Yb^II^ centre shows a close contact with one anthracene C—C bond of a bridging OPhAn ligand, bringing the formal coordination number to five.

## Abstract 2

The crystal structure of a binuclear mixed-valence ytterbium complex containing bridging and terminal 2-anthracene-substituted phenoxide ligands is described. One of the bridging ligands shows a π-inter­action between an anthracene bond and the ytterbium(II) centre.

## Chemical context   

**Figure 1:**
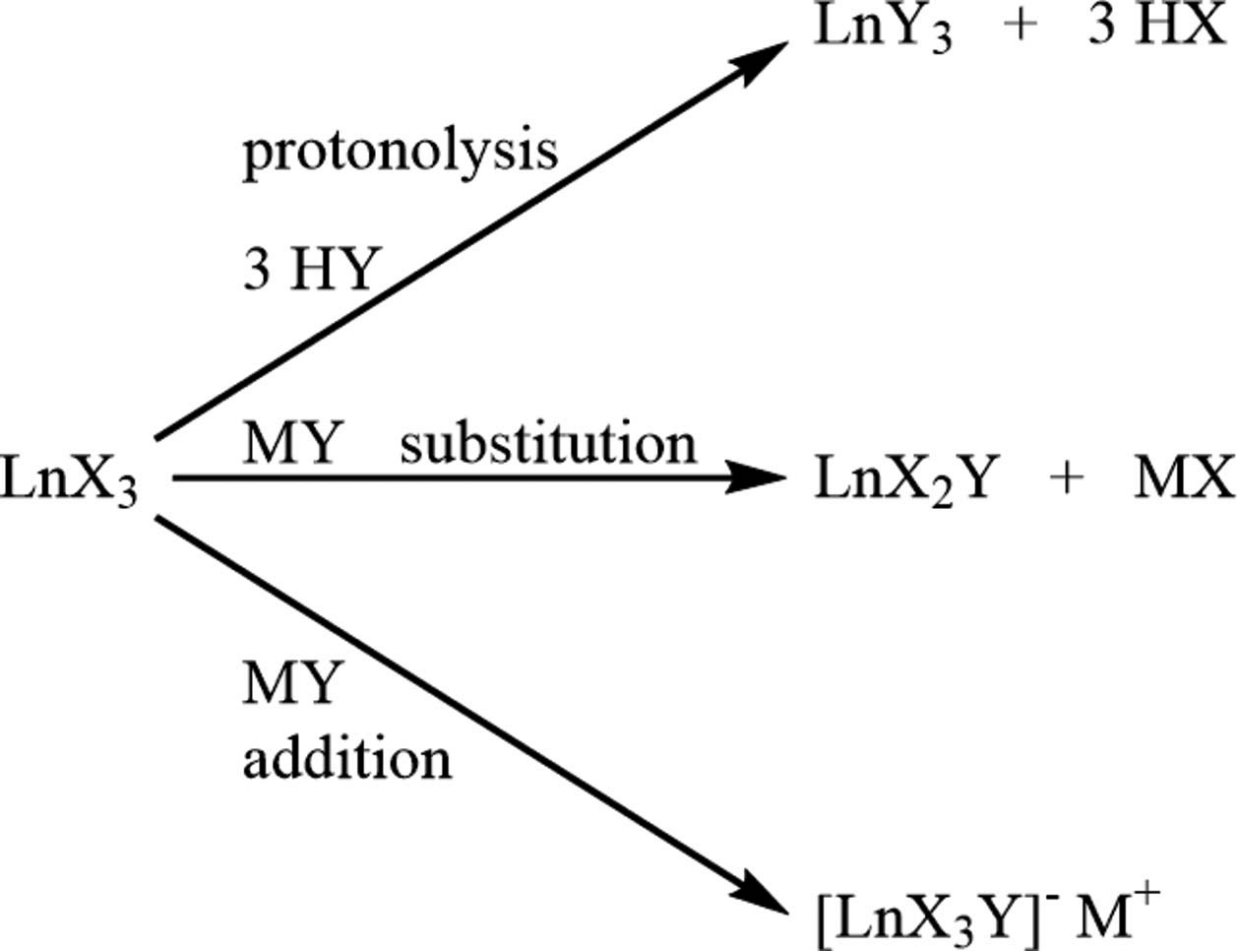
A comparison of the typical outcomes of metathesis with *MY* (*e.g. M* = Na^+^ and *Y* = anionic ligand) *versus* protonolysis with H*Y* (where H*Y* is a stronger acid than H*X*) in lanthanide reactions.

One of the classical methods for introducing ligands into the metal coordination sphere in lanthanide chemistry is by a protonolysis or acid–base reaction that eliminates a suitable protonated ligand from the coordination sphere. The advantage of this method is that it avoids the formation of additional complexes (‘ate’ salt complexes) often favoured during metathesis reactions (Fig. 1 ▸) (Evans, 2000 ▸; Volker *et al.*, 2019 ▸). However, in the case of divalent ytterbium, the metal is a relatively good reducing agent that can lead to competing redox chemistry with the formation of unexpected trivalent ytterbium products (Kagan & Namy, 1984 ▸). We have observed that these redox reactions become more problematic the more acidic the incoming ‘acid’ is. In the case of alcohols and phenols, reduction of the O—H bond is sometimes observed but there are also instances where the product remains divalent (Delbridge *et al.*, 2007 ▸; Binda *et al.*, 2008 ▸). In the case of 2-(anthracen-9-yl)phenol (HOPhAn) discussed here, partial O—H reduction is observed, resulting in a mixed-valence Yb^II^–Yb^III^ dimer featuring bridging aryl­oxides.
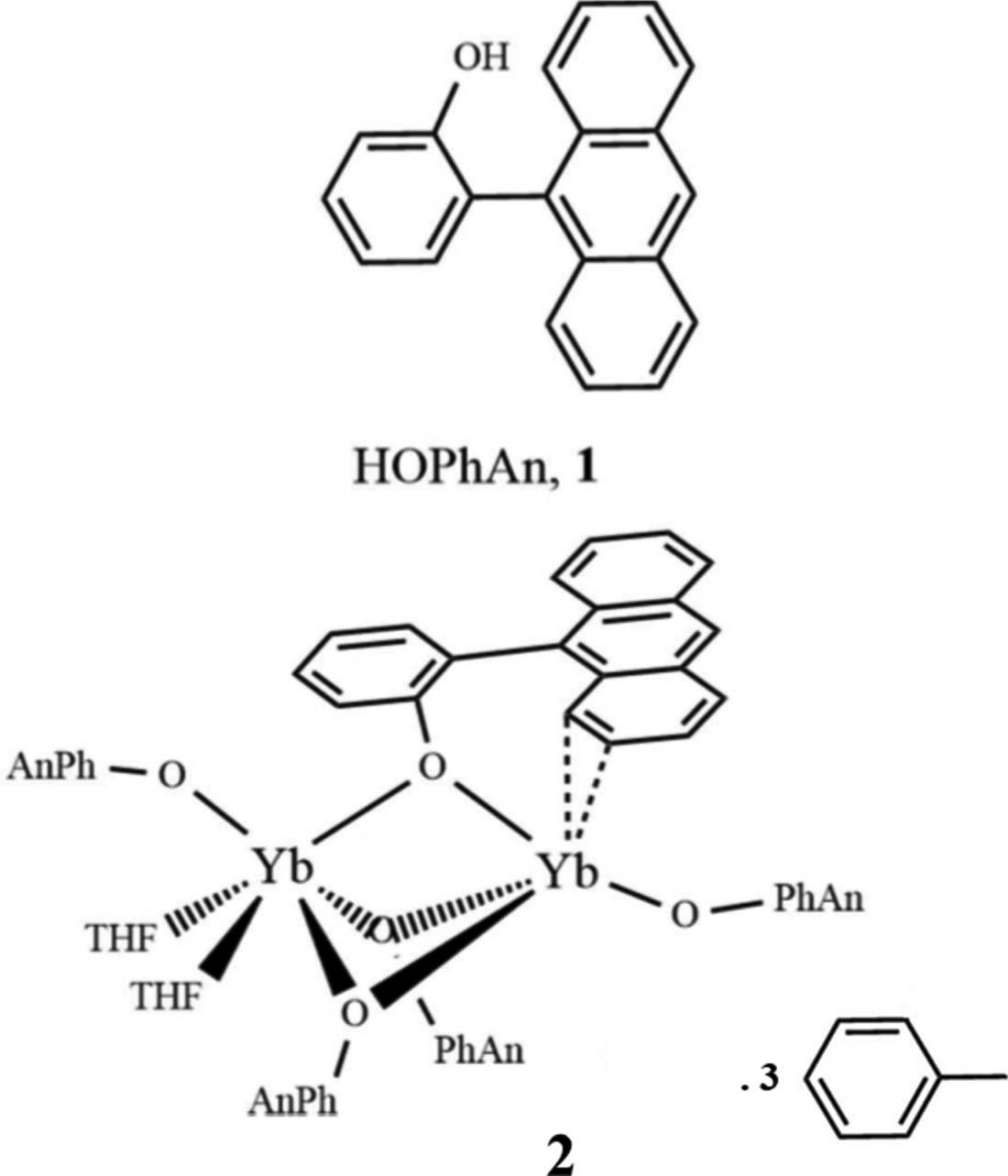


## Structural commentary   

**Figure 2:**
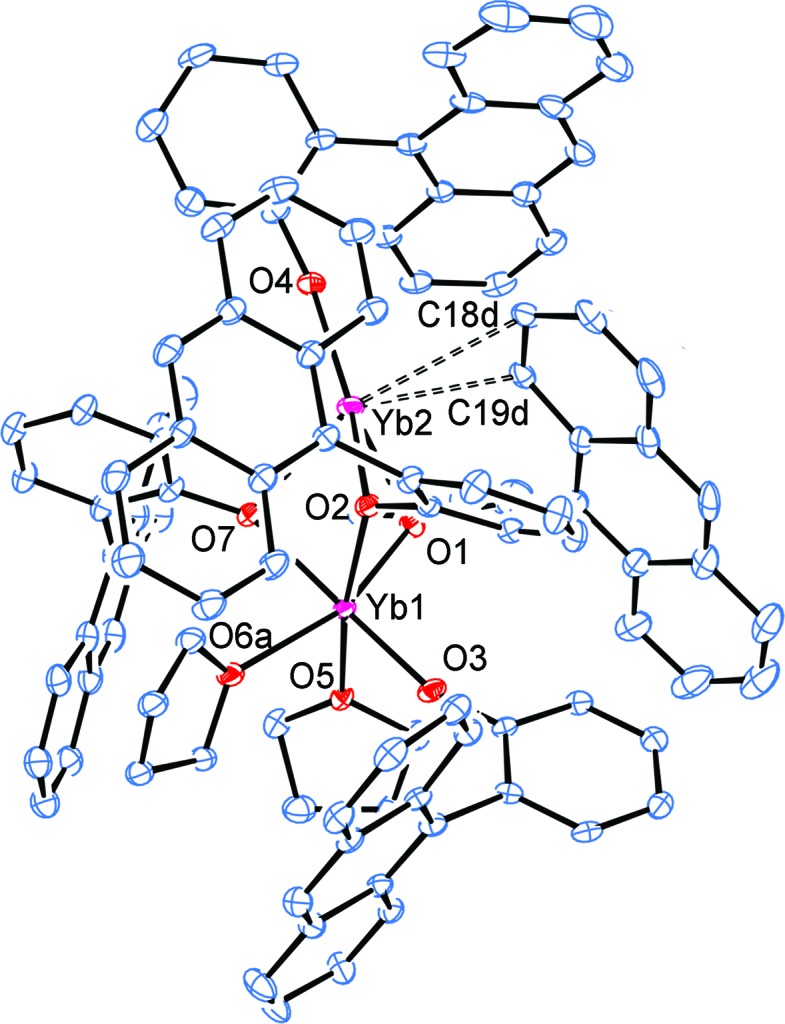
The mol­ecular structure of complex **2**, with partial atom labelling and displacement ellipsoids drawn at the 30% probability level (as also for Figs. 3 ▸ and 4 ▸). For clarity, the toluene mol­ecules of solvation and H atoms have been omitted in this and other figures. Only the major-disorder partner is shown for THF mol­ecule O6*a*/C21*a*–C24*a* and the OPhAn ligand C1*e*–C20*e*.

**Figure 3:**
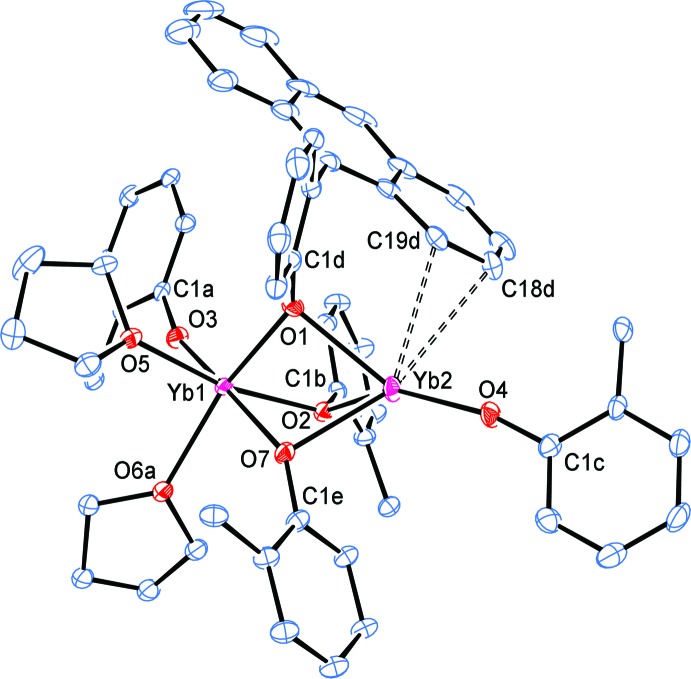
A simplified view of complex **2**, removing all anthracene C atoms except those attached to the phenyl rings or part of the weakly inter­acting anthracene unit.

**Figure 4:**
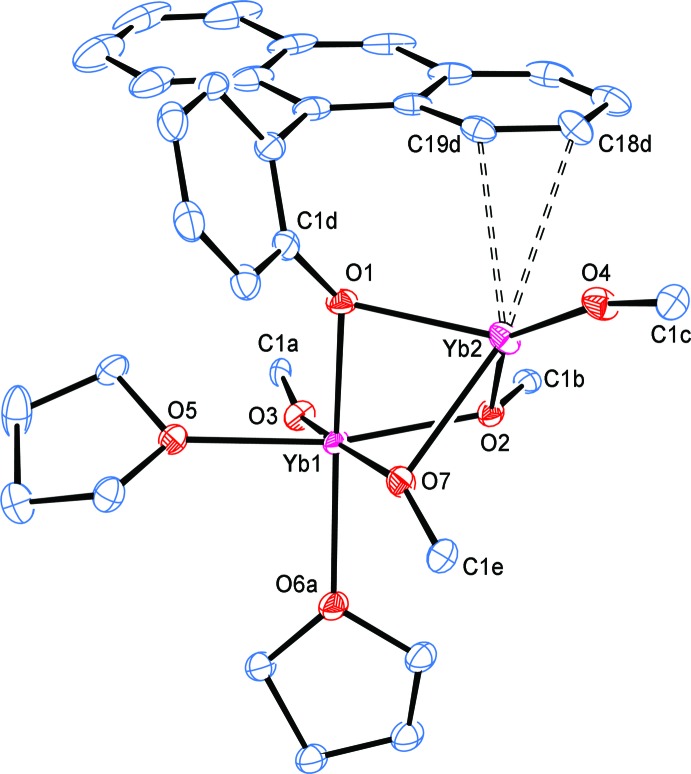
A view of the inner coordination sphere of complex **2**. Only the phenoxide C atom attached to an O atom is shown for all ligands, except for that of the coordinated anthracene group.

The structure of complex **2** (without the three toluenes of solvation) is shown in Fig. 2 ▸. The inner coordination environment is depicted in Figs. 3 ▸ and 4 ▸. In Fig. 3 ▸, the anthracene groups have been removed for clarity, except for the 9-anthracene C atoms. In Fig. 4 ▸, the structure has been further simplified by removing all of the PhAn C atoms except that directly attached to the phenoxide O atom. In both Figs. 3 ▸ and 4 ▸, all the C atoms of the PhAn ligand that show close contacts with Yb2 through atoms C18*d* and C19*d* are depicted.

The two Yb centres in **2** are connected [Yb1⋯Yb2 = 3.2587 (7) Å; Table 1 ▸] by three bridging OPhAn aryl­oxides and each Yb atom is also bound to two terminal aryl­oxides. Atom Yb1 is further coordinated by two THF ligands, bringing the coordination number to 6, while Yb2 shows the aforementioned close contacts with C18*d* and C19*d* for a coordination number of 5 (taking the C18*d*—C19*d* bond as occupying the fifth coordination site). Based on the stoichiometry, **2** is expected to be a Yb^II^/Yb^III^ mixed-valence complex. Selected bond lengths and angles and close contacts between Yb2 and the C atoms of one anthracene group are listed in Table 1 ▸. The substanti­ally shorter terminal and bridging Yb1—OPhAn distances [terminal: 2.077 (3) Å; bridging: 2.191 (3)–2.236 (3) Å] compared to Yb2—OPhAn [terminal: 2.165 (3) Å; bridging: 2.352 (3)–2.416 (3) Å] indicate that Yb1 is trivalent, while Yb2 is divalent.

For ytterbium, the Cambridge Structural Database (CSD, Version 5.38, last update November 2018; Groom *et al.*, 2016 ▸) lists 10 bridging and 88 terminal structures containing nonchelating aryl­oxides. These structures represent both Yb^II^ and Yb^III^ oxidation states but there is only one structure containing a bridging aryl­oxide that contains both Yb^II^ and Yb^III^ in a mixed-valence complex (Deacon *et al.*, 2009 ▸). The supporting information (CSD search S1) lists the CSD refcodes and references of structures containing Yb terminal and bridging nonchelating aryl­oxides used in the structural discussion below.

The terminal and bridging distances for Yb1 compare well with other six-coordinate trivalent Yb aryl­oxides (terminal: 2.023–2.258, median 2.077 Å; bridging: 2.215–2.246, median 2.240 Å).

The terminal and bridging distances for Yb2 also agree fairly well with other five-coordinate divalent Yb aryl­oxides (terminal: 2.168–2.233, median 2.215 Å; bridging: 2.245–2.356, median 2.309 Å).

The terminal Yb2—OPhAn distance is at the short end of the observed range, while the bridging distance is somewhat longer than expected based on literature comparisons (see S1 in the supporting information).

The Yb1—O(THF) distances [2.306 (3) and 2.309 (6) Å] are typical of six-coordinate Yb^III^—O(THF) bond lengths (2.251–2.509 Å; median 2.342 Å) and indicate that the Yb centre is not overly crowded (see CSD search S1 in the supporting information). The most noteworthy feature in the structure of **2** is the presence of a close contact between two anthracene C atoms of one bridging OPhAn ligand and Yb2 [Yb2—C18d = 3.190 (3) Å and Yb2—C19d = 2.970 (3) Å]. This is well within the wide range recognized for weak Yb^II^–arene inter­actions (2.795–3.651 Å, median 2.98 Å) after correction to five-coordination.

It appears that the Yb^II^ centre prefers to satisfy its electron deficiency by coordination of an anthracene π-bond (or the anthracene C—H bonds) rather than coordinating a further mol­ecule of THF.

## Supra­molecular features   

The only noteworthy inter­actions observable in the crystal structure of complex **2** are C—H⋯π inter­actions, both intra- and inter­molecular (see Table S2 in the supporting information). Inter­stitial space is filled with three toluene mol­ecules of solvation (all disordered over two positions; see §5, *Refinement*).

## Synthesis and crystallization   

### Materials and instrumentation   

All solvents were purchased from Sigma–Aldrich Chemicals and dried by distillation from sodium under nitro­gen. The 2-(anthracen-9-yl)phenol was purified by recrystallization from hot toluene, while Yb[N(SiMe)_3_]_2_(THF)_2_ was prepared by analogy to the procedure of Hitchcock *et al.* (2002 ▸) using NaN(SiMe_3_)_2_ and YbI_2_(THF)_*x*_, and recrystallized from hot hexane. NMR spectra were recorded on a Bruker AV III 300 MHz Spectrometer in sealable Teflon-valved tubes and were referenced to residual solvent resonances. The line widths at half maximum (ν_1/2_ in Hz) were measured for all paramagnetic resonances and are reported below. Elemental analyses were performed by Canadian Microanalytical Ltd.

### Synthesis of complex 2   

**Figure 5:**
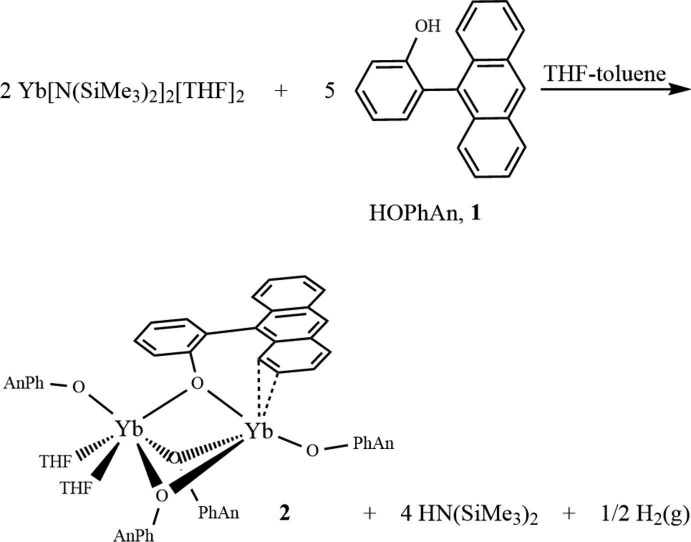
The reaction scheme for the synthesis of complex **2**.

The reaction scheme for the synthesis of complex **2** is illustrated in Fig. 5 ▸. A solution of 2-(anthracen-9-yl)phenol (0.100 g, 0.370 mmol) in THF (10 ml) was prepared in a glove-box and added by Pasteur pipette to a vigorously stirred solution of Yb[N(SiMe)_3_]_2_(THF)_2_ (0.078 g, 0.12 mmol) in toluene (10 ml). The deep-orange solution darkened to red on stirring overnight. The solution was filtered through Celite on a sintered glass frit and the filtrate was evaporated to dryness under reduced pressure. The red solid was recrystallized from a mixture of toluene and hexane at 143 K, yielding deep-orange crystals (yield 0.079 g, 62%). ^1^H NMR (C_6_D_6_, 300 MHz, 296 K): δ 88.4 (6H, ν_1/2_ = 700 Hz), 49.4 (3H, overlaps next resonance), 47.9 (6H, ν_1/2_ = 350 Hz, overlaps previous resonance), 12.86 (2H, ν_1/2_ = 9 Hz), 11.70 (4H, ν_1/2_ = 12 Hz), 10.93 (4H, ν_1/2_ = 12 Hz), 10.00 (4H, ν_1/2_ = 25 Hz), 9.30 (4H, ν_1/2_ = 70 Hz), 1.26 (2H, *t*), 0.96 (2H, *t*), −2.77 (3H, ν_1/2_ = 100 Hz), −3.89 (2H, ν_1/2_ = 14 Hz), −5.38 (2H, ν_1/2_ = 20 Hz), −11.2 (6H, ν_1/2_ ∼ 150 Hz, overlaps next resonance), −11.4 (6H, ν_1/2_ ∼ 300 Hz, overlaps previous resonance), −16.0 (3H, ν_1/2_ = 140 Hz), −24.4 (3H, ν_1/2_ = 800 Hz), −77.2 (3H, ν_1/2_ = 600 Hz). Analysis calculated for C_129_H_105_O_7_Yb_2_ (%): C 73.30, H 5.01; found: C 72.55, H 4.87.

## Refinement   

Crystal data, data collection and structure refinement details are summarized in Table 2 ▸. The C-bound H atoms were included in calculated positions and treated as riding atoms: C—H = 0.95–0.99 Å, with *U*_iso_(H) = 1.5*U*_eq_(C) for methyl H atoms and 1.2*U*_eq_(C) for other H atoms.

One bridging ligand, O7/C1*e*–C20*e*:O7/C1*f*–C20*f*, was disordered and modelled in two positions, with an occupancy ratio of 0.533 (3):0.467 (3), using rigid groups and restraints. A metal-coordinated THF mol­ecule, O6a/C21*a*–C24*a*:O6b/C21*b*–C24*b*, is also disordered and was modelled in two positions, with an occupancy ratio of 0.542 (9):0.458 (9), using restraints. Three toluene solvent mol­ecules are also present and are all disordered over two positions; they were modelled with rigid groups and restraints; the C29 and C43 toluene mol­ecules were modelled at 50% occupancy each and the C36 toluene mol­ecule was modelled with an occupancy ratio of 0.682 (9):0.318 (9).

The largest peak in the final difference electron density synthesis is near atom Yb1 and the largest hole is near atom C19*d*; the r.m.s. deviation is 0.11 e Å^−3^.

## Supplementary Material

Crystal structure: contains datablock(s) Global, I. DOI: 10.1107/S205698901901154X/su5510sup1.cif

Structure factors: contains datablock(s) I. DOI: 10.1107/S205698901901154X/su5510Isup2.hkl

CSD search S1: CSD refcodes and references; C-H...pi interactions: Table S2. DOI: 10.1107/S205698901901154X/su5510sup3.pdf

CCDC reference: 1947717

Additional supporting information:  crystallographic information; 3D view; checkCIF report

## Figures and Tables

**Table 1:** Selected geometric parameters (Å, °)

Yb1—Yb2	3.2587 (7)	Yb2—O1	2.413 (3)
Yb1—O1	2.191 (3)	Yb2—O2	2.352 (3)
Yb1—O2	2.236 (3)	Yb2—O4	2.165 (3)
Yb1—O3	2.077 (3)	Yb2—O7	2.416 (3)
Yb1—O5	2.306 (3)	Yb2—C18*D*	3.190 (3)
Yb1—O6*A*	2.309 (6)	Yb2—C19*D*	2.970 (3)
Yb1—O7	2.229 (3)		
			
O1—Yb1—O2	82.10 (11)	O7—Yb1—O5	103.45 (11)
O1—Yb1—O5	86.34 (12)	O7—Yb1—O6*A*	82.0 (3)
O1—Yb1—O7	77.59 (12)	O1—Yb2—O7	69.99 (10)
O1—Yb1—O6*A*	158.2 (3)	O2—Yb2—O1	75.17 (10)
O2—Yb1—O5	168.04 (11)	O2—Yb2—O7	71.36 (11)
O2—Yb1—O6*A*	100.6 (5)	O4—Yb2—O1	137.75 (12)
O3—Yb1—O1	106.85 (12)	O4—Yb2—O2	147.05 (12)
O3—Yb1—O2	94.92 (12)	O4—Yb2—O7	115.61 (11)
O3—Yb1—O5	85.50 (12)	Yb1—O1—Yb2	89.98 (11)
O3—Yb1—O7	170.39 (12)	Yb1—O2—Yb2	90.47 (11)
O3—Yb1—O6*A*	94.5 (3)	Yb1—O7—Yb2	88.99 (11)
O5—Yb1—O6*A*	91.2 (5)	C1*A*—O3—Yb1	162.0 (3)
O7—Yb1—O2	77.07 (11)	C1*C*—O4—Yb2	166.3 (3)

**Table 2:** Experimental details

Crystal data	
Chemical formula	[Yb_2_(C_20_H_13_O)_5_(C_4_H_8_O)_2_]·3C_7_H_7_
*M*_r_	2113.20
Crystal system, space group	Monoclinic, *P*2_1_/*c*
Temperature (K)	83
*a*, *b*, *c* (Å)	14.4612 (7), 20.8473 (10), 32.0864 (15)
β (°)	95.508 (1)
*V* (Å^3^)	9628.6 (8)
*Z*	4
Radiation type	Mo *K*α
μ (mm^−1^)	1.99
Crystal size (mm)	0.4 × 0.37 × 0.1

Data collection	
Diffractometer	Bruker SMART CCD area detector
Absorption correction	Multi-scan (*SADABS*; Bruker, 2002 ▸)
*T*_min_, *T*_max_	0.729, 0.826
No. of measured, independent and observed [*I* > 2σ(*I*)] reflections	109364, 22107, 15925
*R*_int_	0.067
(sin θ/λ)_max_ (Å^−1^)	0.650

Refinement	
*R*[*F* ^2^ > 2σ(*F* ^2^)], *wR*(*F* ^2^), *S*	0.048, 0.115, 1.03
No. of reflections	22107
No. of parameters	1480
No. of restraints	1509
H-atom treatment	H-atom parameters constrained
Δρ_max_, Δρ_min_ (e Å^−3^)	2.20, −0.93

Computer programs: *SMART* (Bruker, 2002 ▸), *SAINT* (Bruker, 2002 ▸), *SHELXS* (Sheldrick, 2008 ▸), *SHELXL* (Sheldrick, 2015 ▸) and *OLEX2* (Dolomanov *et al.*, 2009 ▸).

## supplementary crystallographic information

### Crystal data

**Table:**

[Yb_2_(C_20_H_13_O)_5_(C_4_H_8_O)_2_]·3C_7_H_7_	*F*(000) = 4300
*M**_r_* = 2113.20	*D*_x_ = 1.458 Mg m^−^^3^
Monoclinic, *P*2_1_/*c*	Mo *K*α radiation, λ = 0.71073 Å
*a* = 14.4612 (7) Å	Cell parameters from 9535 reflections
*b* = 20.8473 (10) Å	θ = 2.3–29.7°
*c* = 32.0864 (15) Å	µ = 1.99 mm^−^^1^
β = 95.508 (1)°	*T* = 83 K
*V* = 9628.6 (8) Å^3^	Fragment, orange
*Z* = 4	0.4 × 0.37 × 0.1 mm

### Data collection

**Table:**

Bruker SMART CCD area detector diffractometer	22107 independent reflections
Parallel, graphite monochromator	15925 reflections with *I* > 2σ(*I*)
Detector resolution: 8.3 pixels mm^-1^	*R*_int_ = 0.067
phi and ω scans	θ_max_ = 27.5°, θ_min_ = 1.4°
Absorption correction: multi-scan (SADABS; Bruker, 2002)	*h* = −18→18
*T*_min_ = 0.729, *T*_max_ = 0.826	*k* = −27→27
109364 measured reflections	*l* = −41→41

### Refinement

**Table:**

Refinement on *F*^2^	Primary atom site location: structure-invariant direct methods
Least-squares matrix: full	Hydrogen site location: inferred from neighbouring sites
*R*[*F*^2^ > 2σ(*F*^2^)] = 0.048	H-atom parameters constrained
*wR*(*F*^2^) = 0.115	*w* = 1/[σ^2^(*F*_o_^2^) + (0.046*P*)^2^ + 19.2482*P*] where *P* = (*F*_o_^2^ + 2*F*_c_^2^)/3
*S* = 1.03	(Δ/σ)_max_ = 0.002
22107 reflections	Δρ_max_ = 2.20 e Å^−^^3^
1480 parameters	Δρ_min_ = −0.93 e Å^−^^3^
1509 restraints	

### Special details

**Table:**

Experimental. The data collection nominally covered a full sphere of reciprocal space by a combination of 5 sets of ω scans each set at different φ and/or 2θ angles and each scan (5 s exposure) covering -0.3° degrees in ω. The crystal to detector distance was 5.035 cm.
Geometry. All esds (except the esd in the dihedral angle between two l.s. planes) are estimated using the full covariance matrix. The cell esds are taken into account individually in the estimation of esds in distances, angles and torsion angles; correlations between esds in cell parameters are only used when they are defined by crystal symmetry. An approximate (isotropic) treatment of cell esds is used for estimating esds involving l.s. planes.
Refinement. One bridging ligand O7-C1E-C20E/O7-C1F-C20F was disordered and modelled in two positions with occupancies 53:47% using rigid groups and restraints (SADI, SIMU). A metal coordinated THF is also disordered and modelled in two positions with occupancy 54:46% using restraints (SADI, SIMU). Three toluene solvent molecules are also present and they are all disordered over two positions and modelled with rigid groups and restraints (SIMU). C29 and C43 toluene molecules were modelled at 50% occupancy each and the C36 toluene was modelled at 68:32%.

### Fractional atomic coordinates and isotropic or equivalent isotropic displacement parameters (Å^2^)

**Table:**

	*x*	*y*	*z*	*U*_iso_*/*U*_eq_	Occ. (<1)
Yb1	0.18124 (2)	0.71235 (2)	0.43346 (2)	0.02267 (6)	
Yb2	0.29037 (2)	0.79160 (2)	0.36561 (2)	0.03018 (6)	
O1	0.1748 (2)	0.70802 (14)	0.36503 (9)	0.0283 (7)	
O2	0.3311 (2)	0.73221 (14)	0.42697 (9)	0.0258 (7)	
O3	0.2033 (2)	0.61927 (14)	0.45529 (10)	0.0299 (7)	
O4	0.3243 (2)	0.87059 (15)	0.32598 (10)	0.0342 (8)	
O5	0.0260 (2)	0.68505 (14)	0.42582 (11)	0.0310 (7)	
O7	0.1808 (2)	0.81575 (14)	0.41572 (11)	0.0316 (7)	
C1A	0.2202 (3)	0.5562 (2)	0.45664 (14)	0.0230 (9)	
C1B	0.4010 (3)	0.6888 (2)	0.43332 (13)	0.0243 (9)	
C1C	0.3547 (3)	0.9243 (2)	0.31022 (15)	0.0312 (11)	
C1D	0.1103 (3)	0.7079 (2)	0.33159 (14)	0.0293 (10)	
C2A	0.2194 (3)	0.5200 (2)	0.42007 (14)	0.0281 (10)	
H2A	0.205598	0.540354	0.393708	0.034*	
C2B	0.3910 (3)	0.6280 (2)	0.41576 (13)	0.0262 (10)	
H2B	0.336306	0.617916	0.398207	0.031*	
C2C	0.3508 (4)	0.9822 (2)	0.33235 (16)	0.0382 (12)	
H2C	0.325719	0.982494	0.358645	0.046*	
C2D	0.0412 (3)	0.7548 (2)	0.32733 (16)	0.0337 (11)	
H2D	0.037827	0.785929	0.348779	0.040*	
C3A	0.2383 (3)	0.4554 (2)	0.42154 (15)	0.0292 (10)	
H3A	0.240208	0.432251	0.396134	0.035*	
C3B	0.4589 (4)	0.5823 (2)	0.42333 (15)	0.0344 (11)	
H3B	0.450623	0.540827	0.411314	0.041*	
C3C	0.3830 (4)	1.0389 (3)	0.31651 (19)	0.0454 (14)	
H3C	0.379386	1.077455	0.332031	0.055*	
C3D	−0.0224 (3)	0.7567 (3)	0.29232 (17)	0.0404 (13)	
H3D	−0.068675	0.789189	0.289812	0.048*	
C4A	0.2543 (3)	0.4237 (2)	0.45887 (16)	0.0297 (10)	
H4A	0.265221	0.378799	0.459615	0.036*	
C4B	0.5393 (4)	0.5966 (2)	0.44839 (17)	0.0398 (13)	
H4B	0.585761	0.564845	0.454417	0.048*	
C4C	0.4201 (4)	1.0401 (3)	0.27849 (18)	0.0483 (15)	
H4C	0.441889	1.079102	0.267623	0.058*	
C4D	−0.0191 (4)	0.7118 (3)	0.26110 (17)	0.0447 (14)	
H4D	−0.063324	0.712957	0.237212	0.054*	
C5A	0.2542 (3)	0.4586 (2)	0.49543 (15)	0.0265 (10)	
H5A	0.264527	0.436775	0.521460	0.032*	
C5B	0.5512 (4)	0.6578 (2)	0.46455 (16)	0.0365 (12)	
H5B	0.606686	0.667661	0.481640	0.044*	
C5C	0.4250 (4)	0.9832 (3)	0.25653 (17)	0.0381 (12)	
H5C	0.451028	0.983682	0.230439	0.046*	
C5D	0.0488 (4)	0.6650 (3)	0.26468 (15)	0.0390 (13)	
H5D	0.051138	0.634067	0.243042	0.047*	
C6A	0.2396 (3)	0.5247 (2)	0.49552 (13)	0.0224 (9)	
C6B	0.4846 (3)	0.7051 (2)	0.45660 (14)	0.0287 (10)	
C6C	0.3934 (3)	0.9256 (2)	0.27131 (15)	0.0296 (10)	
C6D	0.1142 (3)	0.6624 (2)	0.29973 (15)	0.0321 (11)	
C7A	0.2433 (3)	0.5583 (2)	0.53633 (14)	0.0262 (10)	
C7B	0.5042 (3)	0.7732 (2)	0.46942 (15)	0.0278 (10)	
C7C	0.4006 (3)	0.8661 (2)	0.24624 (14)	0.0296 (10)	
C7D	0.1882 (4)	0.6123 (2)	0.30329 (14)	0.0356 (12)	
C8A	0.1610 (3)	0.5697 (2)	0.55534 (14)	0.0254 (10)	
C8B	0.5094 (3)	0.7924 (2)	0.51160 (15)	0.0305 (10)	
C8C	0.4885 (3)	0.8407 (3)	0.23907 (15)	0.0357 (12)	
C8D	0.1647 (4)	0.5464 (2)	0.30659 (15)	0.0424 (14)	
C9A	0.0715 (3)	0.5564 (2)	0.53416 (15)	0.0282 (10)	
H9A	0.067287	0.539731	0.506480	0.034*	
C9B	0.4927 (4)	0.7499 (2)	0.54503 (16)	0.0360 (12)	
H9B	0.479051	0.706123	0.538845	0.043*	
C9C	0.5740 (4)	0.8687 (3)	0.25642 (18)	0.0495 (15)	
H9C	0.573142	0.906888	0.272577	0.059*	
C9D	0.0715 (5)	0.5249 (3)	0.30686 (17)	0.0554 (17)	
H9D	0.022788	0.555632	0.305154	0.067*	
C10A	−0.0073 (3)	0.5668 (2)	0.55266 (17)	0.0370 (12)	
H10A	−0.065892	0.556658	0.538218	0.044*	
C10B	0.4959 (4)	0.7701 (3)	0.58521 (17)	0.0411 (13)	
H10B	0.483752	0.740700	0.606650	0.049*	
C10C	0.6576 (4)	0.8405 (4)	0.2497 (2)	0.070 (2)	
H10C	0.713898	0.859279	0.261676	0.084*	
C10D	0.0501 (6)	0.4618 (3)	0.3095 (2)	0.074 (2)	
H10D	−0.012709	0.448702	0.309959	0.089*	
C11A	−0.0025 (4)	0.5928 (3)	0.59322 (18)	0.0494 (15)	
H11A	−0.058133	0.601387	0.605755	0.059*	
C11B	0.5171 (4)	0.8350 (3)	0.59548 (18)	0.0467 (14)	
H11B	0.519444	0.848887	0.623759	0.056*	
C11C	0.6610 (5)	0.7844 (4)	0.2256 (3)	0.073 (2)	
H11C	0.719516	0.766008	0.221414	0.088*	
C11D	0.1212 (7)	0.4163 (3)	0.3115 (2)	0.079 (3)	
H11D	0.105318	0.372135	0.312091	0.095*	
C12A	0.0811 (4)	0.6056 (3)	0.61464 (17)	0.0460 (14)	
H12A	0.082792	0.622412	0.642221	0.055*	
C12B	0.5342 (4)	0.8771 (2)	0.56509 (17)	0.0408 (13)	
H12B	0.548462	0.920313	0.572544	0.049*	
C12C	0.5837 (5)	0.7568 (3)	0.2085 (2)	0.0595 (18)	
H12C	0.587669	0.719133	0.192080	0.071*	
C12D	0.2109 (7)	0.4327 (3)	0.31252 (18)	0.068 (2)	
H12D	0.257641	0.400583	0.315014	0.082*	
C13A	0.1657 (3)	0.5946 (2)	0.59707 (15)	0.0307 (11)	
C13B	0.5313 (3)	0.8585 (2)	0.52198 (16)	0.0324 (11)	
C13C	0.4941 (4)	0.7833 (3)	0.21442 (17)	0.0423 (13)	
C13D	0.2362 (5)	0.4995 (3)	0.30984 (15)	0.0501 (16)	
C14A	0.2524 (3)	0.6079 (2)	0.61869 (15)	0.0325 (11)	
H14A	0.255160	0.624343	0.646389	0.039*	
C14B	0.5465 (3)	0.9017 (2)	0.49078 (17)	0.0359 (12)	
H14B	0.560763	0.944955	0.498053	0.043*	
C14C	0.4140 (4)	0.7535 (3)	0.19803 (16)	0.0416 (13)	
H14C	0.418457	0.715930	0.181564	0.050*	
C14D	0.3287 (5)	0.5190 (3)	0.31169 (16)	0.0512 (17)	
H14D	0.376095	0.487354	0.314696	0.061*	
C15A	0.3344 (3)	0.5974 (2)	0.60049 (14)	0.0257 (10)	
C15B	0.5416 (3)	0.8834 (2)	0.44901 (16)	0.0322 (11)	
C15C	0.3274 (4)	0.7765 (2)	0.20477 (15)	0.0365 (12)	
C15D	0.3543 (4)	0.5837 (2)	0.30929 (15)	0.0392 (13)	
C16A	0.4228 (3)	0.6083 (2)	0.62278 (16)	0.0334 (11)	
H16A	0.426471	0.622217	0.651074	0.040*	
C16B	0.5574 (3)	0.9278 (2)	0.41616 (18)	0.0393 (13)	
H16B	0.571359	0.971221	0.423074	0.047*	
C16C	0.2436 (4)	0.7443 (3)	0.18899 (16)	0.0401 (13)	
H16C	0.247275	0.707169	0.172046	0.048*	
C16D	0.4480 (4)	0.6040 (3)	0.31152 (16)	0.0469 (15)	
H16D	0.495979	0.572902	0.315808	0.056*	
C17A	0.5018 (3)	0.5989 (2)	0.60417 (16)	0.0348 (12)	
H17A	0.560283	0.605757	0.619681	0.042*	
C17B	0.5527 (4)	0.9092 (3)	0.37576 (18)	0.0459 (14)	
H17B	0.561908	0.939713	0.354555	0.055*	
C17C	0.1596 (4)	0.7658 (3)	0.19780 (16)	0.0405 (13)	
H17C	0.105025	0.743373	0.187391	0.049*	
C17D	0.4714 (4)	0.6670 (3)	0.30769 (16)	0.0410 (13)	
H17D	0.534743	0.679667	0.310128	0.049*	
C18A	0.4984 (3)	0.5790 (2)	0.56188 (17)	0.0338 (11)	
H18A	0.554386	0.574206	0.548931	0.041*	
C18B	0.5342 (4)	0.8442 (3)	0.36484 (17)	0.0393 (12)	
H18B	0.532422	0.831041	0.336419	0.047*	
C18C	0.1522 (4)	0.8218 (2)	0.22252 (15)	0.0351 (11)	
H18C	0.092713	0.836194	0.228779	0.042*	
C18D	0.3992 (3)	0.7138 (2)	0.29995 (16)	0.0363 (12)	
H18D	0.414763	0.757451	0.295968	0.044*	
C19A	0.4149 (3)	0.5667 (2)	0.53958 (15)	0.0285 (10)	
H19A	0.413542	0.553101	0.511270	0.034*	
C19B	0.5190 (3)	0.8005 (2)	0.39515 (16)	0.0343 (11)	
H19B	0.506915	0.757103	0.387344	0.041*	
C19C	0.2288 (3)	0.8547 (2)	0.23718 (14)	0.0299 (10)	
H19C	0.222355	0.892880	0.252793	0.036*	
C19D	0.3086 (3)	0.6960 (2)	0.29828 (14)	0.0310 (11)	
H19D	0.261545	0.727653	0.293317	0.037*	
C20A	0.3301 (3)	0.5740 (2)	0.55821 (14)	0.0245 (9)	
C20B	0.5207 (3)	0.8180 (2)	0.43816 (16)	0.0295 (10)	
C20C	0.3199 (3)	0.8331 (2)	0.22963 (14)	0.0278 (10)	
C20D	0.2824 (4)	0.6306 (2)	0.30381 (13)	0.0314 (11)	
C25	−0.0049 (4)	0.6217 (3)	0.41039 (18)	0.0445 (14)	
H25A	0.040546	0.588411	0.420601	0.053*	
H25B	−0.011492	0.621062	0.379391	0.053*	
C26	−0.0948 (4)	0.6101 (3)	0.4266 (2)	0.0602 (19)	
H26A	−0.089571	0.574751	0.447253	0.072*	
H26B	−0.141968	0.598151	0.403439	0.072*	
C27	−0.1224 (4)	0.6716 (3)	0.44698 (19)	0.0463 (14)	
H27A	−0.188132	0.682369	0.438375	0.056*	
H27B	−0.113473	0.668121	0.477879	0.056*	
C28	−0.0584 (3)	0.7209 (2)	0.43141 (17)	0.0355 (11)	
H28A	−0.085145	0.739666	0.404579	0.043*	
H28B	−0.045856	0.755756	0.452139	0.043*	
C43A	0.2303 (7)	0.2770 (4)	0.3876 (4)	0.078 (3)	0.5
H43A	0.285362	0.280496	0.372109	0.117*	0.5
H43B	0.249717	0.274269	0.417637	0.117*	0.5
H43C	0.191037	0.314970	0.382003	0.117*	0.5
C44A	0.1795 (5)	0.2212 (3)	0.3745 (3)	0.0789 (16)	0.5
C45A	0.0830 (5)	0.2218 (4)	0.3733 (4)	0.0774 (18)	0.5
H45A	0.052084	0.258220	0.383074	0.093*	0.5
C46A	0.0321 (4)	0.1689 (5)	0.3579 (4)	0.0772 (18)	0.5
H46A	−0.033788	0.169338	0.357046	0.093*	0.5
C47A	0.0773 (6)	0.1155 (4)	0.3437 (3)	0.0790 (19)	0.5
H47A	0.042443	0.079426	0.333198	0.095*	0.5
C48A	0.1736 (6)	0.1150 (4)	0.3450 (4)	0.0818 (19)	0.5
H48A	0.204598	0.078473	0.335265	0.098*	0.5
C49A	0.2247 (4)	0.1678 (4)	0.3604 (3)	0.0811 (19)	0.5
H49A	0.290571	0.167309	0.361316	0.097*	0.5
O6A	0.1671 (12)	0.7551 (5)	0.4989 (2)	0.0326 (14)	0.542 (9)
C1E	0.1884 (4)	0.8757 (2)	0.4304 (2)	0.0298 (13)	0.533 (3)
C2E	0.2801 (3)	0.8939 (2)	0.4410 (2)	0.0311 (13)	0.533 (3)
H2E	0.329155	0.864980	0.436773	0.037*	0.533 (3)
C3E	0.3001 (3)	0.9543 (2)	0.4578 (2)	0.0342 (13)	0.533 (3)
H3E	0.362761	0.966742	0.465054	0.041*	0.533 (3)
C4E	0.2283 (4)	0.9966 (2)	0.4640 (2)	0.0366 (13)	0.533 (3)
H4E	0.241967	1.037899	0.475464	0.044*	0.533 (3)
C5E	0.1366 (3)	0.9784 (3)	0.4534 (2)	0.0352 (13)	0.533 (3)
H5E	0.087566	1.007294	0.457594	0.042*	0.533 (3)
C6E	0.1166 (3)	0.9180 (3)	0.4366 (2)	0.0305 (11)	0.533 (3)
C7E	0.0168 (2)	0.8982 (2)	0.42281 (11)	0.0331 (11)	0.533 (3)
C16E	−0.1504 (3)	0.9075 (3)	0.32721 (11)	0.0493 (14)	0.533 (3)
H16E	−0.214191	0.899831	0.318711	0.059*	0.533 (3)
C8E	−0.0443 (2)	0.88209 (19)	0.45364 (10)	0.0342 (11)	0.533 (3)
C9E	−0.0098 (3)	0.8764 (3)	0.49673 (10)	0.0377 (13)	0.533 (3)
H9E	0.053294	0.886347	0.505337	0.045*	0.533 (3)
C10E	−0.0681 (3)	0.8563 (3)	0.52590 (10)	0.0424 (14)	0.533 (3)
H10E	−0.044746	0.852080	0.554468	0.051*	0.533 (3)
C11E	−0.1621 (3)	0.8421 (3)	0.51339 (12)	0.0440 (15)	0.533 (3)
H11E	−0.200939	0.827100	0.533557	0.053*	0.533 (3)
C12E	−0.1984 (3)	0.8497 (3)	0.47227 (13)	0.0414 (13)	0.533 (3)
H12E	−0.262315	0.841122	0.464524	0.050*	0.533 (3)
C13E	−0.1401 (2)	0.87029 (18)	0.44143 (11)	0.0384 (11)	0.533 (3)
C14E	−0.1751 (2)	0.8800 (2)	0.39995 (11)	0.0414 (13)	0.533 (3)
H14E	−0.239550	0.874325	0.392097	0.050*	0.533 (3)
C15E	−0.1172 (2)	0.89793 (19)	0.36992 (10)	0.0422 (12)	0.533 (3)
C17E	−0.0947 (4)	0.9269 (3)	0.29839 (10)	0.0534 (14)	0.533 (3)
H17E	−0.120476	0.935575	0.270603	0.064*	0.533 (3)
C18E	0.0017 (4)	0.9347 (3)	0.30903 (11)	0.0522 (14)	0.533 (3)
H18E	0.040879	0.945904	0.288056	0.063*	0.533 (3)
C19E	0.0380 (3)	0.9261 (3)	0.34878 (12)	0.0460 (12)	0.533 (3)
H19E	0.102745	0.932243	0.355857	0.055*	0.533 (3)
C20E	−0.0206 (2)	0.90768 (18)	0.38094 (10)	0.0386 (12)	0.533 (3)
C21A	0.1062 (17)	0.7393 (8)	0.5294 (8)	0.0336 (16)	0.542 (9)
H21A	0.099630	0.692188	0.531742	0.040*	0.542 (9)
H21B	0.043947	0.758209	0.522054	0.040*	0.542 (9)
C22A	0.1519 (10)	0.7681 (8)	0.5713 (6)	0.0350 (16)	0.542 (9)
H22A	0.118518	0.807237	0.578808	0.042*	0.542 (9)
H22B	0.150809	0.736586	0.594297	0.042*	0.542 (9)
C23A	0.2433 (11)	0.7827 (5)	0.5640 (5)	0.0378 (15)	0.542 (9)
H23A	0.263164	0.823478	0.577952	0.045*	0.542 (9)
H23B	0.285993	0.748202	0.574952	0.045*	0.542 (9)
C24A	0.2441 (7)	0.7882 (5)	0.5211 (3)	0.0358 (14)	0.542 (9)
H24A	0.302918	0.770339	0.512721	0.043*	0.542 (9)
H24B	0.241458	0.834147	0.513299	0.043*	0.542 (9)
C29A	0.8741 (6)	0.0546 (5)	0.4107 (2)	0.054 (2)	0.5
H29A	0.919939	0.022367	0.404242	0.081*	0.5
H29B	0.844884	0.041392	0.435652	0.081*	0.5
H29C	0.905095	0.096032	0.415949	0.081*	0.5
C30A	0.8051 (4)	0.0605 (3)	0.37630 (16)	0.0535 (13)	0.5
C31A	0.8314 (4)	0.0589 (4)	0.33575 (19)	0.0544 (15)	0.5
H31A	0.895366	0.056982	0.331266	0.065*	0.5
C32A	0.7641 (5)	0.0601 (4)	0.30176 (15)	0.0512 (16)	0.5
H32A	0.781986	0.058994	0.274045	0.061*	0.5
C33A	0.6705 (5)	0.0630 (4)	0.30839 (19)	0.0501 (17)	0.5
H33A	0.624526	0.063737	0.285185	0.060*	0.5
C34A	0.6443 (4)	0.0647 (4)	0.3489 (2)	0.0507 (15)	0.5
H34A	0.580405	0.066672	0.353414	0.061*	0.5
C35A	0.7117 (5)	0.0635 (4)	0.38287 (17)	0.0517 (15)	0.5
H35A	0.693817	0.064664	0.410585	0.062*	0.5
O6B	0.1726 (15)	0.7423 (7)	0.5023 (2)	0.0334 (15)	0.458 (9)
C5F	0.1475 (4)	0.9662 (3)	0.4715 (2)	0.0332 (14)	0.467 (3)
H5F	0.098516	0.995934	0.473759	0.040*	0.467 (3)
C6F	0.1324 (4)	0.9115 (3)	0.4470 (2)	0.0316 (12)	0.467 (3)
C1F	0.2041 (5)	0.8679 (3)	0.4437 (2)	0.0313 (13)	0.467 (3)
C2F	0.2909 (4)	0.8790 (3)	0.4650 (2)	0.0311 (13)	0.467 (3)
H2F	0.339930	0.849229	0.462773	0.037*	0.467 (3)
C3F	0.3061 (3)	0.9336 (3)	0.4895 (2)	0.0348 (13)	0.467 (3)
H3F	0.365440	0.941209	0.504077	0.042*	0.467 (3)
C4F	0.2344 (4)	0.9772 (3)	0.4928 (2)	0.0358 (13)	0.467 (3)
H4F	0.244734	1.014562	0.509570	0.043*	0.467 (3)
C7F	0.0397 (2)	0.9035 (3)	0.42304 (12)	0.0324 (11)	0.467 (3)
C8F	0.0316 (3)	0.9164 (2)	0.37963 (12)	0.0395 (13)	0.467 (3)
C9F	0.1117 (3)	0.9351 (3)	0.35855 (15)	0.0434 (16)	0.467 (3)
H9F	0.170698	0.939064	0.374112	0.052*	0.467 (3)
C10F	0.1032 (4)	0.9469 (3)	0.31718 (15)	0.0489 (16)	0.467 (3)
H10F	0.156308	0.958297	0.303532	0.059*	0.467 (3)
C11F	0.0154 (4)	0.9425 (3)	0.29398 (12)	0.0526 (15)	0.467 (3)
H11F	0.009159	0.953431	0.265106	0.063*	0.467 (3)
C12F	−0.0596 (4)	0.9229 (3)	0.31230 (11)	0.0511 (14)	0.467 (3)
H12F	−0.116776	0.917494	0.295496	0.061*	0.467 (3)
C13F	−0.0561 (3)	0.9100 (2)	0.35594 (11)	0.0462 (12)	0.467 (3)
C14F	−0.1340 (2)	0.8919 (3)	0.37520 (12)	0.0431 (13)	0.467 (3)
H14F	−0.192309	0.888457	0.358977	0.052*	0.467 (3)
C15F	−0.1281 (2)	0.8788 (2)	0.41792 (13)	0.0392 (12)	0.467 (3)
C16F	−0.2071 (3)	0.8580 (3)	0.43773 (16)	0.0411 (13)	0.467 (3)
H16F	−0.264952	0.851699	0.421586	0.049*	0.467 (3)
C17F	−0.1994 (3)	0.8471 (3)	0.48043 (16)	0.0423 (13)	0.467 (3)
H17F	−0.251809	0.831962	0.493255	0.051*	0.467 (3)
C18F	−0.1149 (4)	0.8580 (3)	0.50526 (13)	0.0412 (13)	0.467 (3)
H18F	−0.111426	0.851555	0.534677	0.049*	0.467 (3)
C19F	−0.0371 (3)	0.8781 (3)	0.48692 (11)	0.0376 (12)	0.467 (3)
H19F	0.019443	0.885828	0.503775	0.045*	0.467 (3)
C20F	−0.0418 (3)	0.8872 (2)	0.44263 (11)	0.0352 (11)	0.467 (3)
C21B	0.101 (2)	0.7283 (10)	0.5294 (9)	0.0332 (16)	0.458 (9)
H21C	0.093124	0.681387	0.532219	0.040*	0.458 (9)
H21D	0.041345	0.747483	0.518307	0.040*	0.458 (9)
C22B	0.1356 (12)	0.7576 (10)	0.5704 (7)	0.0347 (16)	0.458 (9)
H22C	0.122000	0.729794	0.594090	0.042*	0.458 (9)
H22D	0.107506	0.800380	0.573892	0.042*	0.458 (9)
C23B	0.2472 (13)	0.7632 (7)	0.5672 (5)	0.0372 (15)	0.458 (9)
H23C	0.267779	0.808327	0.570983	0.045*	0.458 (9)
H23D	0.281624	0.736683	0.589046	0.045*	0.458 (9)
C24B	0.2628 (8)	0.7424 (6)	0.5292 (3)	0.0356 (14)	0.458 (9)
H24C	0.289072	0.698481	0.531100	0.043*	0.458 (9)
H24D	0.307895	0.770831	0.516952	0.043*	0.458 (9)
C29B	0.7034 (7)	0.0753 (5)	0.30219 (18)	0.055 (2)	0.5
H29D	0.721217	0.117645	0.292322	0.083*	0.5
H29E	0.635575	0.072810	0.301380	0.083*	0.5
H29F	0.725805	0.042055	0.284074	0.083*	0.5
C30B	0.7433 (5)	0.0659 (3)	0.34387 (16)	0.0528 (13)	0.5
C31B	0.8396 (4)	0.0661 (4)	0.3523 (2)	0.0555 (15)	0.5
H31B	0.877985	0.069540	0.329945	0.067*	0.5
C32B	0.8794 (4)	0.0614 (5)	0.3933 (2)	0.0601 (17)	0.5
H32B	0.945100	0.061571	0.399046	0.072*	0.5
C33B	0.8233 (6)	0.0564 (4)	0.42596 (18)	0.0609 (18)	0.5
H33B	0.850582	0.053133	0.454015	0.073*	0.5
C34B	0.7271 (6)	0.0561 (4)	0.41757 (18)	0.0587 (16)	0.5
H34B	0.688766	0.052728	0.439896	0.070*	0.5
C35B	0.6872 (4)	0.0607 (4)	0.3765 (2)	0.0538 (15)	0.5
H35B	0.621541	0.060394	0.370797	0.065*	0.5
C43B	0.1304 (7)	0.0836 (4)	0.3527 (4)	0.081 (3)	0.5
H43D	0.071015	0.068067	0.361083	0.121*	0.5
H43E	0.181073	0.057203	0.365898	0.121*	0.5
H43F	0.129904	0.080796	0.322143	0.121*	0.5
C44B	0.1438 (5)	0.1484 (3)	0.3654 (3)	0.0777 (16)	0.5
C45B	0.0688 (4)	0.1905 (4)	0.3615 (3)	0.0757 (18)	0.5
H45B	0.010378	0.176639	0.348477	0.091*	0.5
C46B	0.0794 (6)	0.2528 (4)	0.3765 (4)	0.0775 (18)	0.5
H46B	0.028184	0.281606	0.373789	0.093*	0.5
C47B	0.1648 (7)	0.2731 (3)	0.3955 (3)	0.0792 (19)	0.5
H47B	0.172037	0.315759	0.405742	0.095*	0.5
C48B	0.2397 (5)	0.2311 (4)	0.3994 (3)	0.0825 (19)	0.5
H48B	0.298118	0.244925	0.412463	0.099*	0.5
C49B	0.2292 (4)	0.1687 (4)	0.3843 (3)	0.0801 (19)	0.5
H49B	0.280413	0.140009	0.386974	0.096*	0.5
C36B	0.7321 (12)	0.7340 (7)	0.3380 (6)	0.061 (4)	0.318 (9)
H36A	0.690295	0.750905	0.314685	0.091*	0.318 (9)
H36B	0.793497	0.753729	0.337608	0.091*	0.318 (9)
H36C	0.706938	0.743938	0.364511	0.091*	0.318 (9)
C37B	0.7400 (9)	0.6665 (7)	0.3337 (5)	0.0595 (19)	0.318 (9)
C38B	0.6967 (11)	0.6265 (7)	0.3606 (5)	0.0584 (19)	0.318 (9)
H38B	0.665933	0.644285	0.382790	0.070*	0.318 (9)
C39B	0.6986 (13)	0.5604 (7)	0.3550 (6)	0.058 (2)	0.318 (9)
H39B	0.669008	0.533062	0.373378	0.070*	0.318 (9)
C40B	0.7437 (13)	0.5343 (7)	0.3226 (6)	0.060 (2)	0.318 (9)
H40B	0.744901	0.489150	0.318789	0.072*	0.318 (9)
C41B	0.7870 (13)	0.5743 (8)	0.2958 (6)	0.061 (2)	0.318 (9)
H41B	0.817800	0.556526	0.273635	0.073*	0.318 (9)
C42B	0.7852 (11)	0.6404 (8)	0.3014 (5)	0.061 (2)	0.318 (9)
H42B	0.815030	0.667762	0.283170	0.073*	0.318 (9)
C36A	0.8116 (6)	0.5568 (4)	0.2732 (2)	0.071 (3)	0.682 (9)
H36D	0.764253	0.549913	0.249635	0.106*	0.682 (9)
H36E	0.839255	0.515504	0.282132	0.106*	0.682 (9)
H36F	0.860064	0.585252	0.264408	0.106*	0.682 (9)
C37A	0.7700 (4)	0.5852 (3)	0.3070 (2)	0.0593 (18)	0.682 (9)
C38A	0.7716 (5)	0.6516 (3)	0.3114 (2)	0.0612 (18)	0.682 (9)
H38A	0.804067	0.677262	0.293139	0.073*	0.682 (9)
C39A	0.7257 (6)	0.6804 (3)	0.3426 (3)	0.0604 (18)	0.682 (9)
H39A	0.726664	0.725716	0.345658	0.072*	0.682 (9)
C40A	0.6782 (5)	0.6428 (4)	0.3694 (2)	0.0575 (18)	0.682 (9)
H40A	0.646722	0.662541	0.390686	0.069*	0.682 (9)
C41A	0.6767 (6)	0.5765 (4)	0.3649 (2)	0.0573 (19)	0.682 (9)
H41A	0.644326	0.550861	0.383226	0.069*	0.682 (9)
C42A	0.7228 (5)	0.5477 (3)	0.3337 (2)	0.0580 (18)	0.682 (9)
H42A	0.721925	0.502409	0.330759	0.070*	0.682 (9)

### Atomic displacement parameters (Å^2^)

**Table:**

	*U*^11^	*U*^22^	*U*^33^	*U*^12^	*U*^13^	*U*^23^
Yb1	0.02982 (11)	0.01947 (9)	0.01901 (10)	−0.00670 (8)	0.00379 (7)	−0.00032 (8)
Yb2	0.03577 (12)	0.02727 (11)	0.02896 (12)	0.00081 (9)	0.01064 (9)	0.00974 (9)
O1	0.0348 (18)	0.0316 (17)	0.0184 (16)	−0.0016 (14)	0.0016 (13)	−0.0015 (13)
O2	0.0285 (17)	0.0243 (15)	0.0246 (17)	−0.0046 (13)	0.0026 (13)	0.0006 (13)
O3	0.0414 (19)	0.0247 (16)	0.0229 (17)	−0.0058 (14)	−0.0004 (14)	0.0069 (13)
O4	0.047 (2)	0.0305 (17)	0.0273 (18)	−0.0004 (15)	0.0120 (15)	0.0082 (14)
O5	0.0337 (18)	0.0226 (15)	0.037 (2)	−0.0062 (14)	0.0064 (15)	−0.0049 (14)
O7	0.0348 (18)	0.0205 (15)	0.041 (2)	−0.0051 (13)	0.0140 (15)	−0.0025 (14)
C1A	0.021 (2)	0.022 (2)	0.026 (2)	−0.0062 (17)	0.0017 (18)	0.0034 (18)
C1B	0.032 (2)	0.025 (2)	0.016 (2)	−0.0029 (18)	0.0038 (18)	0.0027 (17)
C1C	0.036 (3)	0.028 (2)	0.029 (3)	−0.003 (2)	−0.002 (2)	0.007 (2)
C1D	0.031 (2)	0.036 (3)	0.022 (2)	−0.010 (2)	0.0046 (19)	0.006 (2)
C2A	0.030 (2)	0.032 (2)	0.021 (2)	−0.009 (2)	−0.0021 (19)	−0.0002 (19)
C2B	0.031 (2)	0.029 (2)	0.017 (2)	−0.0043 (19)	−0.0030 (18)	0.0011 (18)
C2C	0.045 (3)	0.042 (3)	0.027 (3)	0.000 (2)	0.001 (2)	0.002 (2)
C2D	0.030 (3)	0.040 (3)	0.032 (3)	−0.005 (2)	0.004 (2)	0.006 (2)
C3A	0.020 (2)	0.034 (2)	0.034 (3)	−0.0101 (19)	0.0027 (19)	−0.008 (2)
C3B	0.042 (3)	0.029 (2)	0.031 (3)	0.000 (2)	−0.003 (2)	−0.007 (2)
C3C	0.053 (4)	0.034 (3)	0.048 (4)	−0.009 (3)	−0.005 (3)	−0.001 (3)
C3D	0.027 (3)	0.054 (3)	0.041 (3)	−0.002 (2)	0.004 (2)	0.010 (3)
C4A	0.019 (2)	0.025 (2)	0.045 (3)	0.0007 (18)	0.003 (2)	0.001 (2)
C4B	0.036 (3)	0.032 (3)	0.049 (3)	0.005 (2)	−0.011 (2)	−0.005 (2)
C4C	0.059 (4)	0.039 (3)	0.046 (4)	−0.024 (3)	0.003 (3)	0.011 (3)
C4D	0.029 (3)	0.073 (4)	0.031 (3)	−0.010 (3)	−0.002 (2)	0.010 (3)
C5A	0.019 (2)	0.030 (2)	0.030 (3)	−0.0036 (18)	−0.0012 (18)	0.013 (2)
C5B	0.034 (3)	0.031 (3)	0.041 (3)	−0.003 (2)	−0.011 (2)	−0.001 (2)
C5C	0.038 (3)	0.045 (3)	0.032 (3)	−0.013 (2)	0.004 (2)	0.009 (2)
C5D	0.042 (3)	0.054 (3)	0.021 (3)	−0.022 (3)	0.006 (2)	−0.006 (2)
C6A	0.017 (2)	0.033 (2)	0.016 (2)	−0.0041 (17)	−0.0027 (16)	0.0038 (18)
C6B	0.035 (3)	0.027 (2)	0.023 (2)	−0.006 (2)	−0.0019 (19)	−0.0021 (19)
C6C	0.030 (3)	0.033 (2)	0.026 (2)	−0.008 (2)	0.001 (2)	0.009 (2)
C6D	0.039 (3)	0.035 (3)	0.023 (2)	−0.010 (2)	0.006 (2)	0.002 (2)
C7A	0.026 (2)	0.028 (2)	0.024 (2)	−0.0073 (18)	−0.0021 (19)	0.0026 (19)
C7B	0.026 (2)	0.026 (2)	0.030 (3)	−0.0026 (18)	−0.0028 (19)	0.0011 (19)
C7C	0.033 (3)	0.037 (3)	0.019 (2)	−0.004 (2)	0.0006 (19)	0.010 (2)
C7D	0.059 (3)	0.031 (3)	0.017 (2)	−0.004 (2)	0.006 (2)	−0.0015 (19)
C8A	0.030 (2)	0.025 (2)	0.021 (2)	−0.0072 (18)	−0.0007 (18)	0.0013 (18)
C8B	0.028 (2)	0.030 (2)	0.032 (3)	−0.005 (2)	−0.004 (2)	−0.002 (2)
C8C	0.031 (3)	0.052 (3)	0.025 (3)	−0.004 (2)	0.004 (2)	0.018 (2)
C8D	0.076 (4)	0.033 (3)	0.018 (2)	−0.004 (3)	0.003 (2)	−0.001 (2)
C9A	0.033 (3)	0.025 (2)	0.027 (2)	−0.0094 (19)	0.000 (2)	−0.0039 (19)
C9B	0.041 (3)	0.030 (3)	0.035 (3)	−0.011 (2)	−0.005 (2)	−0.001 (2)
C9C	0.035 (3)	0.068 (4)	0.046 (3)	−0.004 (3)	0.005 (3)	0.024 (3)
C9D	0.093 (5)	0.049 (3)	0.024 (3)	−0.025 (3)	0.000 (3)	−0.002 (2)
C10A	0.024 (2)	0.044 (3)	0.043 (3)	−0.009 (2)	0.003 (2)	−0.016 (2)
C10B	0.053 (3)	0.038 (3)	0.031 (3)	−0.017 (2)	−0.003 (2)	−0.002 (2)
C10C	0.033 (3)	0.102 (6)	0.076 (5)	−0.005 (4)	0.010 (3)	0.028 (5)
C10D	0.130 (7)	0.053 (4)	0.039 (4)	−0.040 (5)	−0.001 (4)	0.001 (3)
C11A	0.032 (3)	0.071 (4)	0.047 (4)	−0.012 (3)	0.016 (3)	−0.025 (3)
C11B	0.060 (4)	0.044 (3)	0.034 (3)	−0.015 (3)	−0.001 (3)	−0.011 (3)
C11C	0.047 (4)	0.085 (5)	0.093 (6)	0.019 (4)	0.028 (4)	0.026 (5)
C11D	0.159 (9)	0.042 (4)	0.032 (4)	−0.034 (5)	−0.010 (4)	−0.008 (3)
C12A	0.038 (3)	0.067 (4)	0.034 (3)	−0.017 (3)	0.010 (2)	−0.022 (3)
C12B	0.048 (3)	0.030 (3)	0.043 (3)	−0.012 (2)	0.000 (3)	−0.011 (2)
C12C	0.055 (4)	0.062 (4)	0.066 (5)	0.015 (3)	0.029 (3)	0.015 (3)
C12D	0.144 (8)	0.033 (3)	0.025 (3)	−0.001 (4)	−0.002 (4)	−0.002 (2)
C13A	0.030 (3)	0.034 (3)	0.027 (3)	−0.011 (2)	0.002 (2)	−0.008 (2)
C13B	0.031 (3)	0.026 (2)	0.038 (3)	−0.008 (2)	−0.004 (2)	−0.005 (2)
C13C	0.044 (3)	0.054 (3)	0.031 (3)	0.008 (3)	0.015 (2)	0.019 (3)
C13D	0.100 (5)	0.035 (3)	0.014 (2)	0.001 (3)	−0.001 (3)	−0.006 (2)
C14A	0.034 (3)	0.037 (3)	0.026 (3)	−0.008 (2)	−0.003 (2)	−0.006 (2)
C14B	0.030 (3)	0.028 (2)	0.049 (3)	−0.008 (2)	−0.004 (2)	−0.003 (2)
C14C	0.056 (4)	0.045 (3)	0.026 (3)	0.006 (3)	0.016 (2)	0.006 (2)
C14D	0.100 (5)	0.036 (3)	0.018 (3)	0.027 (3)	0.002 (3)	−0.005 (2)
C15A	0.029 (2)	0.022 (2)	0.025 (2)	−0.0066 (18)	−0.0052 (19)	0.0023 (18)
C15B	0.027 (2)	0.030 (2)	0.039 (3)	−0.0036 (19)	−0.001 (2)	0.003 (2)
C15C	0.053 (3)	0.036 (3)	0.021 (2)	0.000 (2)	0.006 (2)	0.010 (2)
C15D	0.061 (4)	0.039 (3)	0.016 (2)	0.018 (3)	0.000 (2)	−0.003 (2)
C16A	0.036 (3)	0.029 (2)	0.032 (3)	−0.008 (2)	−0.011 (2)	−0.002 (2)
C16B	0.036 (3)	0.030 (3)	0.051 (3)	−0.009 (2)	−0.001 (2)	0.007 (2)
C16C	0.062 (4)	0.036 (3)	0.022 (3)	−0.012 (3)	0.003 (2)	0.002 (2)
C16D	0.060 (4)	0.060 (4)	0.021 (3)	0.033 (3)	0.002 (2)	−0.001 (2)
C17A	0.028 (3)	0.030 (2)	0.042 (3)	−0.007 (2)	−0.016 (2)	0.002 (2)
C17B	0.048 (3)	0.047 (3)	0.041 (3)	−0.014 (3)	−0.004 (3)	0.017 (3)
C17C	0.046 (3)	0.044 (3)	0.029 (3)	−0.019 (3)	−0.009 (2)	0.010 (2)
C17D	0.036 (3)	0.061 (4)	0.027 (3)	0.020 (3)	0.005 (2)	0.002 (2)
C18A	0.027 (2)	0.028 (2)	0.045 (3)	−0.0039 (19)	−0.004 (2)	0.002 (2)
C18B	0.041 (3)	0.044 (3)	0.032 (3)	−0.010 (2)	−0.002 (2)	0.006 (2)
C18C	0.034 (3)	0.042 (3)	0.028 (3)	−0.007 (2)	−0.002 (2)	0.012 (2)
C18D	0.037 (3)	0.040 (3)	0.034 (3)	0.007 (2)	0.013 (2)	0.010 (2)
C19A	0.026 (2)	0.028 (2)	0.030 (3)	−0.0038 (19)	−0.0020 (19)	0.000 (2)
C19B	0.031 (3)	0.034 (3)	0.036 (3)	−0.006 (2)	−0.003 (2)	0.002 (2)
C19C	0.035 (3)	0.032 (2)	0.022 (2)	−0.002 (2)	0.000 (2)	0.0094 (19)
C19D	0.037 (3)	0.037 (3)	0.020 (2)	0.009 (2)	0.005 (2)	0.0039 (19)
C20A	0.025 (2)	0.021 (2)	0.027 (2)	−0.0032 (17)	−0.0031 (18)	0.0075 (18)
C20B	0.025 (2)	0.028 (2)	0.035 (3)	−0.0022 (19)	−0.001 (2)	0.000 (2)
C20C	0.034 (3)	0.032 (2)	0.017 (2)	−0.001 (2)	0.0015 (19)	0.0083 (18)
C20D	0.051 (3)	0.030 (2)	0.013 (2)	0.008 (2)	0.002 (2)	0.0004 (18)
C25	0.052 (3)	0.038 (3)	0.044 (3)	−0.020 (3)	0.009 (3)	−0.017 (2)
C26	0.032 (3)	0.029 (3)	0.119 (6)	−0.002 (2)	0.005 (3)	0.001 (3)
C27	0.039 (3)	0.049 (3)	0.053 (4)	−0.003 (3)	0.011 (3)	0.007 (3)
C28	0.033 (3)	0.030 (3)	0.042 (3)	0.000 (2)	−0.002 (2)	0.000 (2)
C43A	0.067 (5)	0.070 (6)	0.099 (6)	0.003 (5)	0.010 (5)	−0.003 (5)
C44A	0.062 (3)	0.075 (4)	0.101 (4)	0.005 (3)	0.010 (3)	−0.012 (3)
C45A	0.060 (3)	0.075 (4)	0.098 (4)	0.006 (3)	0.008 (3)	−0.008 (4)
C46A	0.062 (3)	0.074 (4)	0.096 (4)	0.007 (3)	0.012 (3)	−0.007 (4)
C47A	0.061 (4)	0.076 (4)	0.101 (4)	0.005 (3)	0.011 (3)	−0.009 (4)
C48A	0.062 (4)	0.079 (4)	0.106 (4)	0.005 (3)	0.012 (4)	−0.011 (4)
C49A	0.062 (3)	0.077 (4)	0.104 (4)	0.006 (3)	0.011 (3)	−0.013 (4)
O6A	0.041 (2)	0.030 (4)	0.028 (2)	−0.013 (3)	0.007 (2)	−0.003 (2)
C1E	0.033 (2)	0.021 (2)	0.034 (3)	−0.002 (2)	−0.001 (2)	0.004 (2)
C2E	0.034 (2)	0.021 (2)	0.037 (3)	−0.003 (2)	0.000 (2)	0.001 (2)
C3E	0.038 (3)	0.023 (2)	0.040 (3)	−0.005 (2)	−0.004 (2)	−0.003 (2)
C4E	0.041 (3)	0.024 (2)	0.042 (3)	0.000 (2)	−0.005 (3)	−0.003 (2)
C5E	0.040 (2)	0.024 (2)	0.040 (3)	0.005 (2)	−0.002 (2)	−0.001 (2)
C6E	0.034 (2)	0.021 (2)	0.036 (2)	0.0045 (19)	−0.001 (2)	0.001 (2)
C7E	0.037 (2)	0.0260 (19)	0.036 (2)	0.0073 (19)	−0.002 (2)	−0.0042 (19)
C16E	0.053 (3)	0.050 (3)	0.042 (3)	0.005 (2)	−0.012 (2)	−0.008 (2)
C8E	0.035 (2)	0.027 (2)	0.040 (2)	0.0075 (19)	0.000 (2)	−0.007 (2)
C9E	0.037 (3)	0.032 (2)	0.044 (3)	0.011 (2)	0.006 (2)	−0.003 (2)
C10E	0.040 (3)	0.038 (3)	0.049 (3)	0.010 (3)	0.009 (3)	−0.001 (3)
C11E	0.040 (3)	0.041 (3)	0.054 (3)	0.009 (3)	0.015 (3)	0.001 (3)
C12E	0.036 (2)	0.035 (2)	0.055 (3)	0.006 (2)	0.011 (2)	−0.002 (2)
C13E	0.035 (2)	0.032 (2)	0.049 (2)	0.0080 (19)	0.004 (2)	−0.006 (2)
C14E	0.040 (3)	0.037 (2)	0.047 (3)	0.007 (2)	−0.003 (2)	−0.007 (2)
C15E	0.042 (2)	0.040 (2)	0.042 (2)	0.007 (2)	−0.007 (2)	−0.009 (2)
C17E	0.060 (3)	0.056 (3)	0.041 (3)	0.007 (3)	−0.011 (3)	−0.004 (3)
C18E	0.061 (3)	0.054 (2)	0.040 (3)	0.007 (3)	−0.006 (3)	−0.002 (2)
C19E	0.054 (2)	0.046 (2)	0.036 (2)	0.007 (2)	−0.007 (2)	−0.003 (2)
C20E	0.043 (2)	0.036 (2)	0.035 (2)	0.006 (2)	−0.006 (2)	−0.007 (2)
C21A	0.042 (3)	0.032 (4)	0.028 (2)	−0.011 (3)	0.008 (2)	−0.002 (3)
C22A	0.046 (3)	0.032 (4)	0.028 (2)	−0.011 (3)	0.007 (3)	−0.002 (3)
C23A	0.050 (3)	0.033 (4)	0.031 (2)	−0.012 (3)	0.005 (2)	−0.001 (3)
C24A	0.045 (3)	0.033 (3)	0.030 (2)	−0.013 (3)	0.004 (2)	−0.002 (2)
C29A	0.089 (5)	0.042 (4)	0.032 (5)	0.003 (4)	0.016 (4)	0.004 (4)
C30A	0.083 (3)	0.041 (2)	0.036 (3)	−0.007 (2)	0.006 (2)	0.000 (2)
C31A	0.081 (3)	0.044 (3)	0.038 (3)	−0.001 (3)	0.007 (3)	0.000 (3)
C32A	0.079 (4)	0.041 (3)	0.034 (3)	−0.001 (3)	0.009 (3)	0.002 (3)
C33A	0.077 (4)	0.037 (3)	0.036 (3)	−0.009 (3)	0.002 (3)	0.004 (3)
C34A	0.077 (4)	0.039 (3)	0.036 (3)	−0.010 (3)	0.008 (3)	0.002 (3)
C35A	0.082 (3)	0.040 (3)	0.034 (3)	−0.016 (3)	0.009 (3)	0.000 (3)
O6B	0.041 (2)	0.032 (4)	0.028 (2)	−0.014 (3)	0.007 (2)	−0.002 (2)
C5F	0.037 (3)	0.022 (2)	0.039 (3)	0.006 (2)	−0.001 (3)	−0.001 (2)
C6F	0.035 (2)	0.022 (2)	0.037 (2)	0.003 (2)	0.001 (2)	0.001 (2)
C1F	0.035 (3)	0.021 (2)	0.037 (3)	−0.001 (2)	0.001 (2)	0.003 (2)
C2F	0.034 (3)	0.022 (2)	0.036 (3)	−0.002 (2)	−0.002 (3)	0.001 (2)
C3F	0.038 (3)	0.025 (2)	0.041 (3)	−0.003 (2)	−0.003 (3)	−0.002 (2)
C4F	0.040 (3)	0.024 (2)	0.042 (3)	−0.001 (2)	−0.003 (3)	−0.002 (2)
C7F	0.037 (2)	0.024 (2)	0.035 (2)	0.005 (2)	−0.002 (2)	−0.0050 (19)
C8F	0.046 (2)	0.036 (2)	0.035 (2)	0.009 (2)	−0.005 (2)	−0.006 (2)
C9F	0.052 (3)	0.042 (3)	0.035 (3)	0.010 (3)	−0.004 (3)	−0.004 (3)
C10F	0.058 (3)	0.050 (3)	0.037 (3)	0.008 (3)	−0.005 (3)	−0.002 (3)
C11F	0.062 (3)	0.054 (3)	0.040 (3)	0.009 (3)	−0.010 (3)	−0.001 (3)
C12F	0.057 (3)	0.053 (3)	0.040 (3)	0.009 (3)	−0.013 (3)	−0.004 (2)
C13F	0.052 (2)	0.045 (2)	0.039 (2)	0.008 (2)	−0.009 (2)	−0.006 (2)
C14F	0.044 (2)	0.040 (2)	0.043 (2)	0.006 (2)	−0.009 (2)	−0.008 (2)
C15F	0.038 (2)	0.033 (2)	0.046 (2)	0.009 (2)	−0.001 (2)	−0.007 (2)
C16F	0.035 (2)	0.035 (2)	0.053 (3)	0.006 (2)	0.003 (3)	−0.006 (2)
C17F	0.036 (3)	0.037 (2)	0.055 (3)	0.007 (2)	0.011 (2)	−0.004 (2)
C18F	0.038 (3)	0.035 (2)	0.052 (3)	0.006 (2)	0.009 (2)	−0.004 (2)
C19F	0.035 (2)	0.032 (2)	0.046 (3)	0.006 (2)	0.006 (2)	−0.007 (2)
C20F	0.035 (2)	0.027 (2)	0.043 (2)	0.0072 (19)	0.001 (2)	−0.006 (2)
C21B	0.042 (3)	0.030 (4)	0.028 (2)	−0.012 (3)	0.008 (2)	−0.002 (3)
C22B	0.045 (3)	0.031 (4)	0.029 (2)	−0.012 (3)	0.007 (3)	−0.001 (3)
C23B	0.049 (3)	0.032 (4)	0.031 (3)	−0.011 (3)	0.004 (2)	0.000 (3)
C24B	0.045 (3)	0.032 (3)	0.030 (2)	−0.013 (3)	0.006 (2)	−0.002 (3)
C29B	0.085 (5)	0.045 (4)	0.033 (4)	−0.003 (4)	−0.001 (4)	0.006 (4)
C30B	0.081 (3)	0.041 (2)	0.036 (2)	−0.007 (2)	0.007 (2)	0.001 (2)
C31B	0.082 (3)	0.045 (3)	0.040 (3)	0.000 (3)	0.006 (3)	−0.002 (3)
C32B	0.089 (4)	0.047 (3)	0.044 (3)	−0.001 (3)	0.008 (3)	−0.001 (3)
C33B	0.094 (4)	0.047 (3)	0.042 (3)	−0.005 (3)	0.003 (3)	0.000 (3)
C34B	0.090 (4)	0.046 (3)	0.040 (3)	−0.015 (3)	0.008 (3)	−0.001 (3)
C35B	0.084 (3)	0.042 (3)	0.035 (3)	−0.014 (3)	0.009 (3)	0.000 (3)
C43B	0.054 (5)	0.081 (6)	0.107 (6)	0.003 (5)	0.009 (5)	−0.005 (6)
C44B	0.059 (3)	0.075 (4)	0.099 (4)	0.005 (3)	0.011 (3)	−0.012 (3)
C45B	0.059 (3)	0.073 (4)	0.095 (4)	0.004 (3)	0.010 (3)	−0.009 (3)
C46B	0.060 (3)	0.074 (4)	0.098 (4)	0.005 (3)	0.008 (3)	−0.009 (4)
C47B	0.061 (3)	0.074 (4)	0.102 (4)	0.005 (3)	0.007 (3)	−0.013 (4)
C48B	0.062 (3)	0.079 (4)	0.106 (4)	0.006 (3)	0.007 (3)	−0.014 (4)
C49B	0.060 (3)	0.077 (4)	0.103 (4)	0.007 (3)	0.009 (3)	−0.016 (4)
C36B	0.046 (6)	0.069 (7)	0.067 (7)	−0.018 (6)	0.004 (6)	0.012 (7)
C37B	0.042 (3)	0.076 (4)	0.061 (4)	−0.012 (3)	0.005 (3)	0.009 (3)
C38B	0.040 (3)	0.077 (4)	0.057 (4)	−0.010 (3)	0.001 (3)	0.010 (3)
C39B	0.039 (4)	0.077 (4)	0.056 (4)	−0.010 (3)	0.000 (3)	0.010 (3)
C40B	0.042 (4)	0.078 (4)	0.058 (4)	−0.012 (3)	0.002 (3)	0.008 (3)
C41B	0.044 (3)	0.079 (4)	0.060 (4)	−0.014 (3)	0.006 (3)	0.006 (3)
C42B	0.044 (3)	0.077 (4)	0.061 (4)	−0.013 (3)	0.007 (3)	0.009 (3)
C36A	0.050 (4)	0.089 (5)	0.073 (5)	−0.015 (4)	0.002 (4)	−0.005 (5)
C37A	0.042 (3)	0.077 (4)	0.059 (4)	−0.014 (3)	0.007 (3)	0.005 (3)
C38A	0.045 (3)	0.077 (4)	0.062 (4)	−0.015 (3)	0.008 (3)	0.009 (3)
C39A	0.043 (3)	0.075 (4)	0.063 (4)	−0.011 (3)	0.002 (3)	0.011 (3)
C40A	0.038 (3)	0.078 (4)	0.056 (4)	−0.008 (3)	0.002 (3)	0.010 (3)
C41A	0.039 (3)	0.078 (4)	0.054 (4)	−0.009 (3)	0.000 (3)	0.009 (3)
C42A	0.041 (3)	0.076 (4)	0.056 (4)	−0.011 (3)	0.001 (3)	0.008 (3)

### Geometric parameters (Å, º)

**Table:**

Yb1—Yb2	3.2587 (7)	C45A—H45A	0.9500
Yb1—O1	2.191 (3)	C45A—C46A	1.3894
Yb1—O2	2.236 (3)	C46A—H46A	0.9500
Yb1—O3	2.077 (3)	C46A—C47A	1.3904
Yb1—O5	2.306 (3)	C47A—H47A	0.9500
Yb1—O6A	2.309 (6)	C47A—C48A	1.3896
Yb1—O7	2.229 (3)	C48A—H48A	0.9500
Yb1—O6B	2.310 (7)	C48A—C49A	1.3901
Yb2—O1	2.413 (3)	C49A—H49A	0.9500
Yb2—O2	2.352 (3)	O6A—C21A	1.42 (2)
Yb2—O4	2.165 (3)	O6A—C24A	1.439 (18)
Yb2—O7	2.416 (3)	C1E—C2E	1.3900
Yb2—C18D	3.190 (3)	C1E—C6E	1.3900
Yb2—C19D	2.970 (3)	C2E—H2E	0.9500
O1—C1D	1.353 (5)	C2E—C3E	1.3900
O2—C1B	1.357 (5)	C3E—H3E	0.9500
O3—C1A	1.338 (5)	C3E—C4E	1.3900
O4—C1C	1.320 (5)	C4E—H4E	0.9500
O5—C25	1.464 (5)	C4E—C5E	1.3900
O5—C28	1.457 (6)	C5E—H5E	0.9500
O7—C1E	1.336 (5)	C5E—C6E	1.3900
O7—C1F	1.431 (5)	C6E—C7E	1.526 (5)
C1A—C2A	1.394 (6)	C7E—C8E	1.4280
C1A—C6A	1.414 (6)	C7E—C20E	1.4132
C1B—C2B	1.388 (6)	C16E—H16E	0.9500
C1B—C6B	1.401 (6)	C16E—C15E	1.4220
C1C—C2C	1.405 (7)	C16E—C17E	1.3461
C1C—C6C	1.417 (7)	C8E—C9E	1.4294
C1D—C2D	1.396 (7)	C8E—C13E	1.4241
C1D—C6D	1.399 (7)	C9E—H9E	0.9500
C2A—H2A	0.9500	C9E—C10E	1.3833
C2A—C3A	1.375 (6)	C10E—H10E	0.9500
C2B—H2B	0.9500	C10E—C11E	1.4114
C2B—C3B	1.373 (6)	C11E—H11E	0.9500
C2C—H2C	0.9500	C11E—C12E	1.3817
C2C—C3C	1.384 (7)	C12E—H12E	0.9500
C2D—H2D	0.9500	C12E—C13E	1.4270
C2D—C3D	1.382 (7)	C13E—C14E	1.3925
C3A—H3A	0.9500	C14E—H14E	0.9500
C3A—C4A	1.368 (7)	C14E—C15E	1.3868
C3B—H3B	0.9500	C15E—C20E	1.4215
C3B—C4B	1.381 (7)	C17E—H17E	0.9500
C3C—H3C	0.9500	C17E—C18E	1.4117
C3C—C4C	1.380 (8)	C18E—H18E	0.9500
C3D—H3D	0.9500	C18E—C19E	1.3440
C3D—C4D	1.375 (8)	C19E—H19E	0.9500
C4A—H4A	0.9500	C19E—C20E	1.4494
C4A—C5A	1.380 (7)	C21A—H21A	0.9900
C4B—H4B	0.9500	C21A—H21B	0.9900
C4B—C5B	1.381 (7)	C21A—C22A	1.56 (2)
C4C—H4C	0.9500	C22A—H22A	0.9900
C4C—C5C	1.385 (8)	C22A—H22B	0.9900
C4D—H4D	0.9500	C22A—C23A	1.40 (2)
C4D—C5D	1.382 (8)	C23A—H23A	0.9900
C5A—H5A	0.9500	C23A—H23B	0.9900
C5A—C6A	1.394 (6)	C23A—C24A	1.381 (17)
C5B—H5B	0.9500	C24A—H24A	0.9900
C5B—C6B	1.385 (7)	C24A—H24B	0.9900
C5C—H5C	0.9500	C29A—H29A	0.9800
C5C—C6C	1.384 (6)	C29A—H29B	0.9800
C5D—H5D	0.9500	C29A—H29C	0.9800
C5D—C6D	1.399 (7)	C29A—C30A	1.4201
C6A—C7A	1.481 (6)	C30A—C31A	1.3905
C6B—C7B	1.496 (6)	C30A—C35A	1.3887
C6C—C7C	1.489 (7)	C31A—H31A	0.9500
C6D—C7D	1.493 (7)	C31A—C32A	1.3909
C7A—C8A	1.409 (6)	C32A—H32A	0.9500
C7A—C20A	1.416 (6)	C32A—C33A	1.3908
C7B—C8B	1.407 (7)	C33A—H33A	0.9500
C7B—C20B	1.408 (7)	C33A—C34A	1.3893
C7C—C8C	1.416 (7)	C34A—H34A	0.9500
C7C—C20C	1.415 (6)	C34A—C35A	1.3906
C7D—C8D	1.420 (7)	C35A—H35A	0.9500
C7D—C20D	1.414 (7)	O6B—C21B	1.44 (3)
C8A—C9A	1.430 (6)	O6B—C24B	1.49 (2)
C8A—C13A	1.432 (6)	C5F—H5F	0.9500
C8B—C9B	1.430 (7)	C5F—C6F	1.3900
C8B—C13B	1.446 (6)	C5F—C4F	1.3900
C8C—C9C	1.431 (7)	C6F—C1F	1.3900
C8C—C13C	1.441 (8)	C6F—C7F	1.490 (5)
C8D—C9D	1.421 (9)	C1F—C2F	1.3900
C8D—C13D	1.420 (8)	C2F—H2F	0.9500
C9A—H9A	0.9500	C2F—C3F	1.3900
C9A—C10A	1.352 (7)	C3F—H3F	0.9500
C9B—H9B	0.9500	C3F—C4F	1.3900
C9B—C10B	1.353 (7)	C4F—H4F	0.9500
C9C—H9C	0.9500	C7F—C8F	1.4125
C9C—C10C	1.380 (9)	C7F—C20F	1.4284
C9D—H9D	0.9500	C8F—C9F	1.4501
C9D—C10D	1.356 (8)	C8F—C13F	1.4210
C10A—H10A	0.9500	C9F—H9F	0.9500
C10A—C11A	1.405 (7)	C9F—C10F	1.3440
C10B—H10B	0.9500	C10F—H10F	0.9500
C10B—C11B	1.420 (7)	C10F—C11F	1.4120
C10C—H10C	0.9500	C11F—H11F	0.9500
C10C—C11C	1.406 (11)	C11F—C12F	1.3448
C10D—H10D	0.9500	C12F—H12F	0.9500
C10D—C11D	1.395 (11)	C12F—C13F	1.4220
C11A—H11A	0.9500	C13F—C14F	1.3877
C11A—C12A	1.359 (7)	C14F—H14F	0.9500
C11B—H11B	0.9500	C14F—C15F	1.3922
C11B—C12B	1.352 (8)	C15F—C16F	1.4273
C11C—H11C	0.9500	C15F—C20F	1.4232
C11C—C12C	1.329 (10)	C16F—H16F	0.9500
C11D—H11D	0.9500	C16F—C17F	1.3829
C11D—C12D	1.339 (11)	C17F—H17F	0.9500
C12A—H12A	0.9500	C17F—C18F	1.4120
C12A—C13A	1.413 (7)	C18F—H18F	0.9500
C12B—H12B	0.9500	C18F—C19F	1.3834
C12B—C13B	1.433 (7)	C19F—H19F	0.9500
C12C—H12C	0.9500	C19F—C20F	1.4290
C12C—C13C	1.437 (8)	C21B—H21C	0.9900
C12D—H12D	0.9500	C21B—H21D	0.9900
C12D—C13D	1.443 (8)	C21B—C22B	1.49 (3)
C13A—C14A	1.401 (6)	C22B—H22C	0.9900
C13B—C14B	1.379 (7)	C22B—H22D	0.9900
C13C—C14C	1.374 (8)	C22B—C23B	1.63 (2)
C13D—C14D	1.394 (9)	C23B—H23C	0.9900
C14A—H14A	0.9500	C23B—H23D	0.9900
C14A—C15A	1.388 (7)	C23B—C24B	1.33 (2)
C14B—H14B	0.9500	C24B—H24C	0.9900
C14B—C15B	1.388 (7)	C24B—H24D	0.9900
C14C—H14C	0.9500	C29B—H29D	0.9800
C14C—C15C	1.378 (7)	C29B—H29E	0.9800
C14D—H14D	0.9500	C29B—H29F	0.9800
C14D—C15D	1.403 (8)	C29B—C30B	1.4186
C15A—C16A	1.422 (6)	C30B—C31B	1.3922
C15A—C20A	1.438 (6)	C30B—C35B	1.3897
C15B—C16B	1.436 (7)	C31B—H31B	0.9500
C15B—C20B	1.432 (6)	C31B—C32B	1.3893
C15C—C16C	1.434 (7)	C32B—H32B	0.9500
C15C—C20C	1.434 (7)	C32B—C33B	1.3898
C15D—C16D	1.414 (8)	C33B—H33B	0.9500
C15D—C20D	1.426 (7)	C33B—C34B	1.3904
C16A—H16A	0.9500	C34B—H34B	0.9500
C16A—C17A	1.353 (7)	C34B—C35B	1.3896
C16B—H16B	0.9500	C35B—H35B	0.9500
C16B—C17B	1.349 (8)	C43B—H43D	0.9800
C16C—H16C	0.9500	C43B—H43E	0.9800
C16C—C17C	1.350 (8)	C43B—H43F	0.9800
C16D—H16D	0.9500	C43B—C44B	1.4189
C16D—C17D	1.364 (8)	C44B—C45B	1.3923
C17A—H17A	0.9500	C44B—C49B	1.3889
C17A—C18A	1.415 (7)	C45B—H45B	0.9500
C17B—H17B	0.9500	C45B—C46B	1.3896
C17B—C18B	1.419 (7)	C46B—H46B	0.9500
C17C—H17C	0.9500	C46B—C47B	1.3906
C17C—C18C	1.421 (8)	C47B—H47B	0.9500
C17D—H17D	0.9500	C47B—C48B	1.3899
C17D—C18D	1.433 (7)	C48B—H48B	0.9500
C18A—H18A	0.9500	C48B—C49B	1.3907
C18A—C19A	1.367 (6)	C49B—H49B	0.9500
C18B—H18B	0.9500	C36B—H36A	0.9800
C18B—C19B	1.366 (7)	C36B—H36B	0.9800
C18C—H18C	0.9500	C36B—H36C	0.9800
C18C—C19C	1.349 (7)	C36B—C37B	1.4191
C18D—H18D	0.9500	C37B—C38B	1.3924
C18D—C19D	1.358 (7)	C37B—C42B	1.3880
C19A—H19A	0.9500	C38B—H38B	0.9500
C19A—C20A	1.424 (6)	C38B—C39B	1.3890
C19B—H19B	0.9500	C39B—H39B	0.9500
C19B—C20B	1.425 (7)	C39B—C40B	1.3911
C19C—H19C	0.9500	C40B—H40B	0.9500
C19C—C20C	1.435 (7)	C40B—C41B	1.3898
C19D—H19D	0.9500	C41B—H41B	0.9500
C19D—C20D	1.430 (7)	C41B—C42B	1.3902
C25—H25A	0.9900	C42B—H42B	0.9500
C25—H25B	0.9900	C36A—H36D	0.9800
C25—C26	1.466 (8)	C36A—H36E	0.9800
C26—H26A	0.9900	C36A—H36F	0.9800
C26—H26B	0.9900	C36A—C37A	1.4192
C26—C27	1.511 (8)	C37A—C38A	1.3922
C27—H27A	0.9900	C37A—C42A	1.3883
C27—H27B	0.9900	C38A—H38A	0.9500
C27—C28	1.501 (7)	C38A—C39A	1.3896
C28—H28A	0.9900	C39A—H39A	0.9500
C28—H28B	0.9900	C39A—C40A	1.3910
C43A—H43A	0.9800	C40A—H40A	0.9500
C43A—H43B	0.9800	C40A—C41A	1.3900
C43A—H43C	0.9800	C41A—H41A	0.9500
C43A—C44A	1.4193	C41A—C42A	1.3904
C44A—C45A	1.3920	C42A—H42A	0.9500
C44A—C49A	1.3888		
			
O1—Yb1—O2	82.10 (11)	C45A—C44A—C43A	119.3
O1—Yb1—O5	86.34 (12)	C49A—C44A—C43A	120.5
O1—Yb1—O7	77.59 (12)	C49A—C44A—C45A	120.0
O1—Yb1—O6A	158.2 (3)	C44A—C45A—H45A	120.0
O1—Yb1—O6B	165.6 (4)	C46A—C45A—C44A	119.9
O2—Yb1—O5	168.04 (11)	C46A—C45A—H45A	120.0
O2—Yb1—O6A	100.6 (5)	C45A—C46A—H46A	120.0
O2—Yb1—O6B	100.4 (6)	C45A—C46A—C47A	120.1
O3—Yb1—O1	106.85 (12)	C47A—C46A—H46A	120.0
O3—Yb1—O2	94.92 (12)	C46A—C47A—H47A	120.0
O3—Yb1—O5	85.50 (12)	C48A—C47A—C46A	120.0
O3—Yb1—O7	170.39 (12)	C48A—C47A—H47A	120.0
O3—Yb1—O6A	94.5 (3)	C47A—C48A—H48A	120.0
O3—Yb1—O6B	87.2 (4)	C47A—C48A—C49A	120.0
O5—Yb1—O6A	91.2 (5)	C49A—C48A—H48A	120.0
O5—Yb1—O6B	91.6 (6)	C44A—C49A—C48A	120.0
O7—Yb1—O2	77.07 (11)	C44A—C49A—H49A	120.0
O7—Yb1—O5	103.45 (11)	C48A—C49A—H49A	120.0
O7—Yb1—O6A	82.0 (3)	C21A—O6A—Yb1	130.8 (10)
O7—Yb1—O6B	89.1 (4)	C21A—O6A—C24A	105.8 (10)
O1—Yb2—O7	69.99 (10)	C24A—O6A—Yb1	120.3 (9)
O2—Yb2—O1	75.17 (10)	O7—C1E—C2E	112.7 (4)
O2—Yb2—O7	71.36 (11)	O7—C1E—C6E	127.3 (4)
O4—Yb2—O1	137.75 (12)	C2E—C1E—C6E	120.0
O4—Yb2—O2	147.05 (12)	C1E—C2E—H2E	120.0
O4—Yb2—O7	115.61 (11)	C3E—C2E—C1E	120.0
Yb1—O1—Yb2	89.98 (11)	C3E—C2E—H2E	120.0
Yb1—O2—Yb2	90.47 (11)	C2E—C3E—H3E	120.0
Yb1—O7—Yb2	88.99 (11)	C2E—C3E—C4E	120.0
C1D—O1—Yb1	139.0 (3)	C4E—C3E—H3E	120.0
C1D—O1—Yb2	115.6 (3)	C3E—C4E—H4E	120.0
C1B—O2—Yb1	125.2 (3)	C5E—C4E—C3E	120.0
C1B—O2—Yb2	126.8 (3)	C5E—C4E—H4E	120.0
C1A—O3—Yb1	162.0 (3)	C4E—C5E—H5E	120.0
C1C—O4—Yb2	166.3 (3)	C4E—C5E—C6E	120.0
C25—O5—Yb1	121.4 (3)	C6E—C5E—H5E	120.0
C28—O5—Yb1	132.8 (3)	C1E—C6E—C7E	119.1 (4)
C28—O5—C25	105.8 (4)	C5E—C6E—C1E	120.0
C1E—O7—Yb1	144.7 (4)	C5E—C6E—C7E	120.9 (4)
C1E—O7—Yb2	113.1 (3)	C8E—C7E—C6E	119.6 (3)
C1F—O7—Yb1	125.5 (4)	C20E—C7E—C6E	120.1 (3)
C1F—O7—Yb2	116.5 (4)	C20E—C7E—C8E	119.1
O3—C1A—C2A	121.2 (4)	C15E—C16E—H16E	118.8
O3—C1A—C6A	120.3 (4)	C17E—C16E—H16E	118.8
C2A—C1A—C6A	118.5 (4)	C17E—C16E—C15E	122.3
O2—C1B—C2B	120.0 (4)	C7E—C8E—C9E	120.5
O2—C1B—C6B	120.7 (4)	C13E—C8E—C7E	120.0
C2B—C1B—C6B	119.2 (4)	C13E—C8E—C9E	119.5
O4—C1C—C2C	120.3 (5)	C8E—C9E—H9E	120.0
O4—C1C—C6C	121.9 (4)	C10E—C9E—C8E	120.0
C2C—C1C—C6C	117.8 (4)	C10E—C9E—H9E	120.0
O1—C1D—C2D	120.6 (4)	C9E—C10E—H10E	119.9
O1—C1D—C6D	120.5 (4)	C9E—C10E—C11E	120.3
C2D—C1D—C6D	118.8 (4)	C11E—C10E—H10E	119.9
C1A—C2A—H2A	119.5	C10E—C11E—H11E	119.5
C3A—C2A—C1A	121.0 (4)	C12E—C11E—C10E	121.0
C3A—C2A—H2A	119.5	C12E—C11E—H11E	119.5
C1B—C2B—H2B	119.4	C11E—C12E—H12E	120.0
C3B—C2B—C1B	121.1 (4)	C11E—C12E—C13E	120.0
C3B—C2B—H2B	119.4	C13E—C12E—H12E	120.0
C1C—C2C—H2C	119.4	C8E—C13E—C12E	119.0
C3C—C2C—C1C	121.1 (5)	C14E—C13E—C8E	119.5
C3C—C2C—H2C	119.4	C14E—C13E—C12E	121.5
C1D—C2D—H2D	119.6	C13E—C14E—H14E	119.5
C3D—C2D—C1D	120.9 (5)	C15E—C14E—C13E	121.0
C3D—C2D—H2D	119.6	C15E—C14E—H14E	119.5
C2A—C3A—H3A	119.4	C14E—C15E—C16E	122.5
C4A—C3A—C2A	121.3 (5)	C14E—C15E—C20E	120.6
C4A—C3A—H3A	119.4	C20E—C15E—C16E	116.9
C2B—C3B—H3B	120.0	C16E—C17E—H17E	119.7
C2B—C3B—C4B	120.0 (4)	C16E—C17E—C18E	120.7
C4B—C3B—H3B	120.0	C18E—C17E—H17E	119.7
C2C—C3C—H3C	119.5	C17E—C18E—H18E	119.9
C4C—C3C—C2C	120.9 (5)	C19E—C18E—C17E	120.1
C4C—C3C—H3C	119.5	C19E—C18E—H18E	119.9
C2D—C3D—H3D	119.8	C18E—C19E—H19E	119.7
C4D—C3D—C2D	120.4 (5)	C18E—C19E—C20E	120.5
C4D—C3D—H3D	119.8	C20E—C19E—H19E	119.7
C3A—C4A—H4A	120.8	C7E—C20E—C15E	119.6
C3A—C4A—C5A	118.5 (4)	C7E—C20E—C19E	121.2
C5A—C4A—H4A	120.8	C15E—C20E—C19E	119.3
C3B—C4B—H4B	120.5	O6A—C21A—H21A	110.7
C3B—C4B—C5B	119.0 (5)	O6A—C21A—H21B	110.7
C5B—C4B—H4B	120.5	O6A—C21A—C22A	105.4 (13)
C3C—C4C—H4C	120.7	H21A—C21A—H21B	108.8
C3C—C4C—C5C	118.5 (5)	C22A—C21A—H21A	110.7
C5C—C4C—H4C	120.7	C22A—C21A—H21B	110.7
C3D—C4D—H4D	120.2	C21A—C22A—H22A	110.6
C3D—C4D—C5D	119.6 (5)	C21A—C22A—H22B	110.6
C5D—C4D—H4D	120.2	H22A—C22A—H22B	108.7
C4A—C5A—H5A	118.8	C23A—C22A—C21A	105.7 (14)
C4A—C5A—C6A	122.3 (4)	C23A—C22A—H22A	110.6
C6A—C5A—H5A	118.8	C23A—C22A—H22B	110.6
C4B—C5B—H5B	119.0	C22A—C23A—H23A	110.5
C4B—C5B—C6B	122.0 (5)	C22A—C23A—H23B	110.5
C6B—C5B—H5B	119.0	H23A—C23A—H23B	108.6
C4C—C5C—H5C	118.9	C24A—C23A—C22A	106.4 (13)
C6C—C5C—C4C	122.2 (5)	C24A—C23A—H23A	110.5
C6C—C5C—H5C	118.9	C24A—C23A—H23B	110.5
C4D—C5D—H5D	119.6	O6A—C24A—H24A	109.2
C4D—C5D—C6D	120.9 (5)	O6A—C24A—H24B	109.2
C6D—C5D—H5D	119.6	C23A—C24A—O6A	112.0 (10)
C1A—C6A—C7A	123.2 (4)	C23A—C24A—H24A	109.2
C5A—C6A—C1A	118.3 (4)	C23A—C24A—H24B	109.2
C5A—C6A—C7A	118.4 (4)	H24A—C24A—H24B	107.9
C1B—C6B—C7B	120.5 (4)	H29A—C29A—H29B	109.5
C5B—C6B—C1B	118.3 (4)	H29A—C29A—H29C	109.5
C5B—C6B—C7B	121.0 (4)	H29B—C29A—H29C	109.5
C1C—C6C—C7C	120.9 (4)	C30A—C29A—H29A	109.5
C5C—C6C—C1C	119.4 (5)	C30A—C29A—H29B	109.5
C5C—C6C—C7C	119.7 (4)	C30A—C29A—H29C	109.5
C1D—C6D—C7D	119.8 (4)	C31A—C30A—C29A	119.4
C5D—C6D—C1D	119.4 (5)	C35A—C30A—C29A	120.5
C5D—C6D—C7D	120.8 (5)	C35A—C30A—C31A	120.0
C8A—C7A—C6A	120.2 (4)	C30A—C31A—H31A	120.0
C8A—C7A—C20A	119.4 (4)	C30A—C31A—C32A	120.0
C20A—C7A—C6A	120.2 (4)	C32A—C31A—H31A	120.0
C8B—C7B—C6B	121.7 (4)	C31A—C32A—H32A	120.0
C8B—C7B—C20B	119.9 (4)	C33A—C32A—C31A	119.9
C20B—C7B—C6B	118.3 (4)	C33A—C32A—H32A	120.0
C8C—C7C—C6C	120.7 (4)	C32A—C33A—H33A	120.0
C20C—C7C—C6C	120.7 (4)	C34A—C33A—C32A	120.1
C20C—C7C—C8C	118.6 (5)	C34A—C33A—H33A	120.0
C8D—C7D—C6D	120.5 (5)	C33A—C34A—H34A	120.0
C20D—C7D—C6D	119.6 (4)	C33A—C34A—C35A	119.9
C20D—C7D—C8D	119.8 (5)	C35A—C34A—H34A	120.0
C7A—C8A—C9A	121.7 (4)	C30A—C35A—C34A	120.0
C7A—C8A—C13A	120.0 (4)	C30A—C35A—H35A	120.0
C9A—C8A—C13A	118.3 (4)	C34A—C35A—H35A	120.0
C7B—C8B—C9B	123.2 (4)	C21B—O6B—Yb1	129.0 (14)
C7B—C8B—C13B	118.9 (4)	C21B—O6B—C24B	106.7 (13)
C9B—C8B—C13B	117.9 (4)	C24B—O6B—Yb1	115.4 (10)
C7C—C8C—C9C	122.6 (5)	C6F—C5F—H5F	120.0
C7C—C8C—C13C	119.9 (5)	C6F—C5F—C4F	120.0
C9C—C8C—C13C	117.5 (5)	C4F—C5F—H5F	120.0
C7D—C8D—C9D	122.6 (6)	C5F—C6F—C7F	118.0 (5)
C13D—C8D—C7D	119.6 (6)	C1F—C6F—C5F	120.0
C13D—C8D—C9D	117.8 (5)	C1F—C6F—C7F	121.9 (5)
C8A—C9A—H9A	119.3	C6F—C1F—O7	114.3 (5)
C10A—C9A—C8A	121.5 (4)	C2F—C1F—O7	125.6 (5)
C10A—C9A—H9A	119.3	C2F—C1F—C6F	120.0
C8B—C9B—H9B	119.0	C1F—C2F—H2F	120.0
C10B—C9B—C8B	121.9 (5)	C1F—C2F—C3F	120.0
C10B—C9B—H9B	119.0	C3F—C2F—H2F	120.0
C8C—C9C—H9C	119.9	C2F—C3F—H3F	120.0
C10C—C9C—C8C	120.1 (7)	C2F—C3F—C4F	120.0
C10C—C9C—H9C	119.9	C4F—C3F—H3F	120.0
C8D—C9D—H9D	119.0	C5F—C4F—H4F	120.0
C10D—C9D—C8D	122.0 (7)	C3F—C4F—C5F	120.0
C10D—C9D—H9D	119.0	C3F—C4F—H4F	120.0
C9A—C10A—H10A	120.0	C8F—C7F—C6F	118.2 (4)
C9A—C10A—C11A	120.0 (5)	C8F—C7F—C20F	119.0
C11A—C10A—H10A	120.0	C20F—C7F—C6F	122.6 (4)
C9B—C10B—H10B	119.8	C7F—C8F—C9F	121.1
C9B—C10B—C11B	120.3 (5)	C7F—C8F—C13F	119.6
C11B—C10B—H10B	119.8	C13F—C8F—C9F	119.2
C9C—C10C—H10C	119.4	C8F—C9F—H9F	119.7
C9C—C10C—C11C	121.2 (7)	C10F—C9F—C8F	120.6
C11C—C10C—H10C	119.4	C10F—C9F—H9F	119.7
C9D—C10D—H10D	120.3	C9F—C10F—H10F	120.0
C9D—C10D—C11D	119.4 (8)	C9F—C10F—C11F	120.1
C11D—C10D—H10D	120.3	C11F—C10F—H10F	120.0
C10A—C11A—H11A	119.8	C10F—C11F—H11F	119.6
C12A—C11A—C10A	120.4 (5)	C12F—C11F—C10F	120.7
C12A—C11A—H11A	119.8	C12F—C11F—H11F	119.6
C10B—C11B—H11B	119.9	C11F—C12F—H12F	118.8
C12B—C11B—C10B	120.1 (5)	C11F—C12F—C13F	122.3
C12B—C11B—H11B	119.9	C13F—C12F—H12F	118.8
C10C—C11C—H11C	119.5	C8F—C13F—C12F	117.0
C12C—C11C—C10C	121.0 (7)	C14F—C13F—C8F	120.5
C12C—C11C—H11C	119.5	C14F—C13F—C12F	122.5
C10D—C11D—H11D	118.8	C13F—C14F—H14F	119.5
C12D—C11D—C10D	122.4 (7)	C13F—C14F—C15F	121.0
C12D—C11D—H11D	118.8	C15F—C14F—H14F	119.5
C11A—C12A—H12A	119.0	C14F—C15F—C16F	121.5
C11A—C12A—C13A	121.9 (5)	C14F—C15F—C20F	119.5
C13A—C12A—H12A	119.0	C20F—C15F—C16F	119.1
C11B—C12B—H12B	119.0	C15F—C16F—H16F	120.0
C11B—C12B—C13B	122.1 (5)	C17F—C16F—C15F	120.0
C13B—C12B—H12B	119.0	C17F—C16F—H16F	120.0
C11C—C12C—H12C	119.6	C16F—C17F—H17F	119.5
C11C—C12C—C13C	120.8 (7)	C16F—C17F—C18F	121.0
C13C—C12C—H12C	119.6	C18F—C17F—H17F	119.5
C11D—C12D—H12D	120.2	C17F—C18F—H18F	119.9
C11D—C12D—C13D	119.7 (7)	C19F—C18F—C17F	120.3
C13D—C12D—H12D	120.2	C19F—C18F—H18F	119.9
C12A—C13A—C8A	117.8 (4)	C18F—C19F—H19F	120.0
C14A—C13A—C8A	119.6 (4)	C18F—C19F—C20F	120.0
C14A—C13A—C12A	122.6 (5)	C20F—C19F—H19F	120.0
C12B—C13B—C8B	117.7 (4)	C7F—C20F—C19F	120.4
C14B—C13B—C8B	120.1 (5)	C15F—C20F—C7F	120.0
C14B—C13B—C12B	122.2 (4)	C15F—C20F—C19F	119.5
C12C—C13C—C8C	119.4 (6)	O6B—C21B—H21C	110.8
C14C—C13C—C8C	119.7 (5)	O6B—C21B—H21D	110.8
C14C—C13C—C12C	120.9 (6)	O6B—C21B—C22B	104.7 (15)
C8D—C13D—C12D	118.8 (7)	H21C—C21B—H21D	108.9
C14D—C13D—C8D	119.4 (5)	C22B—C21B—H21C	110.8
C14D—C13D—C12D	121.8 (6)	C22B—C21B—H21D	110.8
C13A—C14A—H14A	119.3	C21B—C22B—H22C	111.2
C15A—C14A—C13A	121.4 (4)	C21B—C22B—H22D	111.2
C15A—C14A—H14A	119.3	C21B—C22B—C23B	102.6 (16)
C13B—C14B—H14B	119.2	H22C—C22B—H22D	109.2
C13B—C14B—C15B	121.7 (4)	C23B—C22B—H22C	111.2
C15B—C14B—H14B	119.2	C23B—C22B—H22D	111.2
C13C—C14C—H14C	119.0	C22B—C23B—H23C	110.3
C13C—C14C—C15C	121.9 (5)	C22B—C23B—H23D	110.3
C15C—C14C—H14C	119.0	H23C—C23B—H23D	108.6
C13D—C14D—H14D	118.8	C24B—C23B—C22B	106.9 (13)
C13D—C14D—C15D	122.4 (5)	C24B—C23B—H23C	110.3
C15D—C14D—H14D	118.8	C24B—C23B—H23D	110.3
C14A—C15A—C16A	121.8 (4)	O6B—C24B—H24C	110.0
C14A—C15A—C20A	119.3 (4)	O6B—C24B—H24D	110.0
C16A—C15A—C20A	118.9 (4)	C23B—C24B—O6B	108.2 (12)
C14B—C15B—C16B	122.3 (5)	C23B—C24B—H24C	110.0
C14B—C15B—C20B	119.0 (4)	C23B—C24B—H24D	110.0
C20B—C15B—C16B	118.6 (5)	H24C—C24B—H24D	108.4
C14C—C15C—C16C	122.1 (5)	H29D—C29B—H29E	109.5
C14C—C15C—C20C	119.5 (5)	H29D—C29B—H29F	109.5
C20C—C15C—C16C	118.4 (5)	H29E—C29B—H29F	109.5
C14D—C15D—C16D	122.8 (5)	C30B—C29B—H29D	109.5
C14D—C15D—C20D	118.2 (6)	C30B—C29B—H29E	109.5
C16D—C15D—C20D	119.0 (5)	C30B—C29B—H29F	109.5
C15A—C16A—H16A	119.6	C31B—C30B—C29B	119.4
C17A—C16A—C15A	120.8 (5)	C35B—C30B—C29B	120.5
C17A—C16A—H16A	119.6	C35B—C30B—C31B	120.0
C15B—C16B—H16B	119.2	C30B—C31B—H31B	120.0
C17B—C16B—C15B	121.6 (5)	C32B—C31B—C30B	119.9
C17B—C16B—H16B	119.2	C32B—C31B—H31B	120.0
C15C—C16C—H16C	119.4	C31B—C32B—H32B	120.0
C17C—C16C—C15C	121.1 (5)	C31B—C32B—C33B	120.1
C17C—C16C—H16C	119.4	C33B—C32B—H32B	120.0
C15D—C16D—H16D	119.1	C32B—C33B—H33B	120.0
C17D—C16D—C15D	121.9 (5)	C32B—C33B—C34B	120.0
C17D—C16D—H16D	119.1	C34B—C33B—H33B	120.0
C16A—C17A—H17A	119.6	C33B—C34B—H34B	120.0
C16A—C17A—C18A	120.8 (4)	C35B—C34B—C33B	120.0
C18A—C17A—H17A	119.6	C35B—C34B—H34B	120.0
C16B—C17B—H17B	119.9	C30B—C35B—H35B	120.0
C16B—C17B—C18B	120.2 (5)	C34B—C35B—C30B	120.0
C18B—C17B—H17B	119.9	C34B—C35B—H35B	120.0
C16C—C17C—H17C	119.7	H43D—C43B—H43E	109.5
C16C—C17C—C18C	120.5 (5)	H43D—C43B—H43F	109.5
C18C—C17C—H17C	119.7	H43E—C43B—H43F	109.5
C16D—C17D—H17D	120.4	C44B—C43B—H43D	109.5
C16D—C17D—C18D	119.2 (5)	C44B—C43B—H43E	109.5
C18D—C17D—H17D	120.4	C44B—C43B—H43F	109.5
C17A—C18A—H18A	119.9	C45B—C44B—C43B	119.4
C19A—C18A—C17A	120.3 (5)	C49B—C44B—C43B	120.5
C19A—C18A—H18A	119.9	C49B—C44B—C45B	120.0
C17B—C18B—H18B	120.0	C44B—C45B—H45B	120.0
C19B—C18B—C17B	120.0 (5)	C46B—C45B—C44B	120.0
C19B—C18B—H18B	120.0	C46B—C45B—H45B	120.0
C17C—C18C—H18C	119.7	C45B—C46B—H46B	120.0
C19C—C18C—C17C	120.6 (5)	C45B—C46B—C47B	120.0
C19C—C18C—H18C	119.7	C47B—C46B—H46B	120.0
C17D—C18D—H18D	119.8	C46B—C47B—H47B	120.0
C19D—C18D—C17D	120.4 (5)	C48B—C47B—C46B	120.0
C19D—C18D—H18D	119.8	C48B—C47B—H47B	120.0
C18A—C19A—H19A	119.6	C47B—C48B—H48B	120.0
C18A—C19A—C20A	120.9 (5)	C47B—C48B—C49B	119.9
C20A—C19A—H19A	119.6	C49B—C48B—H48B	120.0
C18B—C19B—H19B	119.0	C44B—C49B—C48B	120.1
C18B—C19B—C20B	122.0 (5)	C44B—C49B—H49B	120.0
C20B—C19B—H19B	119.0	C48B—C49B—H49B	120.0
C18C—C19C—H19C	119.4	H36A—C36B—H36B	109.5
C18C—C19C—C20C	121.2 (5)	H36A—C36B—H36C	109.5
C20C—C19C—H19C	119.4	H36B—C36B—H36C	109.5
C18D—C19D—H19D	119.3	C37B—C36B—H36A	109.5
C18D—C19D—C20D	121.4 (5)	C37B—C36B—H36B	109.5
C20D—C19D—H19D	119.3	C37B—C36B—H36C	109.5
C7A—C20A—C15A	120.1 (4)	C38B—C37B—C36B	119.3
C7A—C20A—C19A	121.7 (4)	C42B—C37B—C36B	120.6
C19A—C20A—C15A	118.2 (4)	C42B—C37B—C38B	120.0
C7B—C20B—C15B	120.3 (4)	C37B—C38B—H38B	120.0
C7B—C20B—C19B	122.1 (4)	C39B—C38B—C37B	119.9
C19B—C20B—C15B	117.6 (4)	C39B—C38B—H38B	120.0
C7C—C20C—C15C	120.4 (5)	C38B—C39B—H39B	120.0
C7C—C20C—C19C	121.4 (4)	C38B—C39B—C40B	120.0
C15C—C20C—C19C	118.2 (4)	C40B—C39B—H39B	120.0
C7D—C20D—C15D	120.4 (5)	C39B—C40B—H40B	120.0
C7D—C20D—C19D	121.5 (4)	C41B—C40B—C39B	120.0
C15D—C20D—C19D	118.1 (5)	C41B—C40B—H40B	120.0
O5—C25—H25A	110.5	C40B—C41B—H41B	120.0
O5—C25—H25B	110.5	C40B—C41B—C42B	119.9
O5—C25—C26	106.4 (4)	C42B—C41B—H41B	120.0
H25A—C25—H25B	108.6	C37B—C42B—C41B	120.1
C26—C25—H25A	110.5	C37B—C42B—H42B	120.0
C26—C25—H25B	110.5	C41B—C42B—H42B	120.0
C25—C26—H26A	110.3	H36D—C36A—H36E	109.5
C25—C26—H26B	110.3	H36D—C36A—H36F	109.5
C25—C26—C27	107.1 (4)	H36E—C36A—H36F	109.5
H26A—C26—H26B	108.6	C37A—C36A—H36D	109.5
C27—C26—H26A	110.3	C37A—C36A—H36E	109.5
C27—C26—H26B	110.3	C37A—C36A—H36F	109.5
C26—C27—H27A	111.0	C38A—C37A—C36A	119.3
C26—C27—H27B	111.0	C42A—C37A—C36A	120.5
H27A—C27—H27B	109.0	C42A—C37A—C38A	120.0
C28—C27—C26	103.9 (5)	C37A—C38A—H38A	120.0
C28—C27—H27A	111.0	C39A—C38A—C37A	119.9
C28—C27—H27B	111.0	C39A—C38A—H38A	120.0
O5—C28—C27	103.9 (4)	C38A—C39A—H39A	120.0
O5—C28—H28A	111.0	C38A—C39A—C40A	120.0
O5—C28—H28B	111.0	C40A—C39A—H39A	120.0
C27—C28—H28A	111.0	C39A—C40A—H40A	120.0
C27—C28—H28B	111.0	C41A—C40A—C39A	120.0
H28A—C28—H28B	109.0	C41A—C40A—H40A	120.0
H43A—C43A—H43B	109.5	C40A—C41A—H41A	120.0
H43A—C43A—H43C	109.5	C40A—C41A—C42A	120.0
H43B—C43A—H43C	109.5	C42A—C41A—H41A	120.0
C44A—C43A—H43A	109.5	C37A—C42A—C41A	120.1
C44A—C43A—H43B	109.5	C37A—C42A—H42A	120.0
C44A—C43A—H43C	109.5	C41A—C42A—H42A	120.0
			
Yb1—O1—C1D—C2D	51.1 (6)	C16A—C15A—C20A—C19A	−4.1 (6)
Yb1—O1—C1D—C6D	−131.7 (4)	C16A—C17A—C18A—C19A	−2.4 (7)
Yb1—O2—C1B—C2B	46.8 (5)	C16B—C15B—C20B—C7B	−179.6 (4)
Yb1—O2—C1B—C6B	−134.9 (4)	C16B—C15B—C20B—C19B	1.5 (7)
Yb1—O3—C1A—C2A	−10.8 (12)	C16B—C17B—C18B—C19B	1.4 (8)
Yb1—O3—C1A—C6A	169.0 (7)	C16C—C15C—C20C—C7C	179.3 (4)
Yb1—O5—C25—C26	155.3 (4)	C16C—C15C—C20C—C19C	−0.4 (6)
Yb1—O5—C28—C27	−146.7 (4)	C16C—C17C—C18C—C19C	0.7 (7)
Yb1—O7—C1E—C2E	80.0 (7)	C16D—C15D—C20D—C7D	−177.2 (4)
Yb1—O7—C1E—C6E	−98.2 (6)	C16D—C15D—C20D—C19D	3.7 (6)
Yb1—O7—C1F—C6F	−118.8 (4)	C16D—C17D—C18D—C19D	2.9 (8)
Yb1—O7—C1F—C2F	64.8 (7)	C17A—C18A—C19A—C20A	0.4 (7)
Yb1—O6A—C21A—C22A	161.4 (11)	C17B—C18B—C19B—C20B	0.2 (8)
Yb1—O6A—C24A—C23A	−150.2 (8)	C17C—C18C—C19C—C20C	−2.3 (7)
Yb1—O6B—C21B—C22B	177.6 (12)	C17D—C18D—C19D—C20D	−0.5 (7)
Yb1—O6B—C24B—C23B	177.3 (8)	C18A—C19A—C20A—C7A	−176.6 (4)
Yb2—O1—C1D—C2D	−72.2 (5)	C18A—C19A—C20A—C15A	2.8 (6)
Yb2—O1—C1D—C6D	104.9 (4)	C18B—C19B—C20B—C7B	179.4 (5)
Yb2—O2—C1B—C2B	−76.0 (5)	C18B—C19B—C20B—C15B	−1.7 (7)
Yb2—O2—C1B—C6B	102.3 (4)	C18C—C19C—C20C—C7C	−177.6 (4)
Yb2—O4—C1C—C2C	−40.5 (16)	C18C—C19C—C20C—C15C	2.1 (6)
Yb2—O4—C1C—C6C	138.5 (12)	C18D—C19D—C20D—C7D	178.2 (5)
Yb2—O7—C1E—C2E	−44.8 (5)	C18D—C19D—C20D—C15D	−2.8 (7)
Yb2—O7—C1E—C6E	137.0 (4)	C20A—C7A—C8A—C9A	178.3 (4)
Yb2—O7—C1F—C6F	131.8 (3)	C20A—C7A—C8A—C13A	−2.5 (6)
Yb2—O7—C1F—C2F	−44.6 (6)	C20A—C15A—C16A—C17A	2.3 (7)
O1—C1D—C2D—C3D	177.1 (4)	C20B—C7B—C8B—C9B	179.4 (5)
O1—C1D—C6D—C5D	−177.4 (4)	C20B—C7B—C8B—C13B	0.3 (7)
O1—C1D—C6D—C7D	2.1 (7)	C20B—C15B—C16B—C17B	0.1 (8)
O2—C1B—C2B—C3B	−176.9 (4)	C20C—C7C—C8C—C9C	−176.7 (4)
O2—C1B—C6B—C5B	175.7 (4)	C20C—C7C—C8C—C13C	1.5 (7)
O2—C1B—C6B—C7B	−9.3 (7)	C20C—C15C—C16C—C17C	−1.1 (7)
O3—C1A—C2A—C3A	178.9 (4)	C20D—C7D—C8D—C9D	179.1 (5)
O3—C1A—C6A—C5A	178.2 (4)	C20D—C7D—C8D—C13D	−0.8 (7)
O3—C1A—C6A—C7A	−0.5 (6)	C20D—C15D—C16D—C17D	−1.4 (7)
O4—C1C—C2C—C3C	179.5 (5)	C25—O5—C28—C27	36.8 (5)
O4—C1C—C6C—C5C	−179.3 (5)	C25—C26—C27—C28	14.6 (7)
O4—C1C—C6C—C7C	0.5 (7)	C26—C27—C28—O5	−31.3 (6)
O5—C25—C26—C27	7.5 (7)	C28—O5—C25—C26	−27.7 (6)
O7—C1E—C2E—C3E	−178.3 (6)	C43A—C44A—C45A—C46A	175.6
O7—C1E—C6E—C5E	178.1 (7)	C43A—C44A—C49A—C48A	−175.5
O7—C1E—C6E—C7E	−4.7 (6)	C44A—C45A—C46A—C47A	0.1
O7—C1F—C2F—C3F	176.3 (7)	C45A—C44A—C49A—C48A	0.2
C1A—C2A—C3A—C4A	3.0 (7)	C45A—C46A—C47A—C48A	0.0
C1A—C6A—C7A—C8A	83.9 (6)	C46A—C47A—C48A—C49A	0.0
C1A—C6A—C7A—C20A	−101.7 (5)	C47A—C48A—C49A—C44A	−0.2
C1B—C2B—C3B—C4B	−0.7 (8)	C49A—C44A—C45A—C46A	−0.2
C1B—C6B—C7B—C8B	112.4 (5)	O6A—C21A—C22A—C23A	−14.7 (16)
C1B—C6B—C7B—C20B	−69.7 (6)	C1E—C2E—C3E—C4E	0.0
C1C—C2C—C3C—C4C	−0.2 (8)	C1E—C6E—C7E—C8E	107.6 (4)
C1C—C6C—C7C—C8C	−114.8 (5)	C1E—C6E—C7E—C20E	−84.8 (4)
C1C—C6C—C7C—C20C	64.2 (6)	C2E—C1E—C6E—C5E	0.0
C1D—C2D—C3D—C4D	0.5 (8)	C2E—C1E—C6E—C7E	177.2 (6)
C1D—C6D—C7D—C8D	115.4 (5)	C2E—C3E—C4E—C5E	0.0
C1D—C6D—C7D—C20D	−63.9 (6)	C3E—C4E—C5E—C6E	0.0
C2A—C1A—C6A—C5A	−2.0 (6)	C4E—C5E—C6E—C1E	0.0
C2A—C1A—C6A—C7A	179.3 (4)	C4E—C5E—C6E—C7E	−177.1 (6)
C2A—C3A—C4A—C5A	−2.1 (7)	C5E—C6E—C7E—C8E	−75.2 (5)
C2B—C1B—C6B—C5B	−6.0 (7)	C5E—C6E—C7E—C20E	92.3 (5)
C2B—C1B—C6B—C7B	169.0 (4)	C6E—C1E—C2E—C3E	0.0
C2B—C3B—C4B—C5B	−1.9 (8)	C6E—C7E—C8E—C9E	−8.1 (4)
C2C—C1C—C6C—C5C	−0.4 (7)	C6E—C7E—C8E—C13E	173.3 (4)
C2C—C1C—C6C—C7C	179.4 (4)	C6E—C7E—C20E—C15E	−170.8 (4)
C2C—C3C—C4C—C5C	−0.3 (9)	C6E—C7E—C20E—C19E	10.3 (4)
C2D—C1D—C6D—C5D	−0.3 (7)	C7E—C8E—C9E—C10E	−175.4
C2D—C1D—C6D—C7D	179.2 (4)	C7E—C8E—C13E—C12E	175.4
C2D—C3D—C4D—C5D	−0.6 (8)	C7E—C8E—C13E—C14E	−5.6
C3A—C4A—C5A—C6A	−0.8 (7)	C16E—C15E—C20E—C7E	−179.2
C3B—C4B—C5B—C6B	0.4 (9)	C16E—C15E—C20E—C19E	−0.3
C3C—C4C—C5C—C6C	0.5 (9)	C16E—C17E—C18E—C19E	−3.8
C3D—C4D—C5D—C6D	0.2 (8)	C8E—C7E—C20E—C15E	−3.2
C4A—C5A—C6A—C1A	2.9 (6)	C8E—C7E—C20E—C19E	177.9
C4A—C5A—C6A—C7A	−178.4 (4)	C8E—C9E—C10E—C11E	−0.7
C4B—C5B—C6B—C1B	3.6 (8)	C8E—C13E—C14E—C15E	3.3
C4B—C5B—C6B—C7B	−171.5 (5)	C9E—C8E—C13E—C12E	−3.3
C4C—C5C—C6C—C1C	−0.2 (8)	C9E—C8E—C13E—C14E	175.7
C4C—C5C—C6C—C7C	−180.0 (5)	C9E—C10E—C11E—C12E	−1.9
C4D—C5D—C6D—C1D	0.2 (7)	C10E—C11E—C12E—C13E	1.9
C4D—C5D—C6D—C7D	−179.3 (5)	C11E—C12E—C13E—C8E	0.7
C5A—C6A—C7A—C8A	−94.8 (5)	C11E—C12E—C13E—C14E	−178.3
C5A—C6A—C7A—C20A	79.6 (5)	C12E—C13E—C14E—C15E	−177.7
C5B—C6B—C7B—C8B	−72.7 (7)	C13E—C8E—C9E—C10E	3.3
C5B—C6B—C7B—C20B	105.2 (5)	C13E—C14E—C15E—C16E	179.2
C5C—C6C—C7C—C8C	65.0 (6)	C13E—C14E—C15E—C20E	−0.9
C5C—C6C—C7C—C20C	−116.0 (5)	C14E—C15E—C20E—C7E	0.9
C5D—C6D—C7D—C8D	−65.1 (6)	C14E—C15E—C20E—C19E	179.8
C5D—C6D—C7D—C20D	115.6 (5)	C15E—C16E—C17E—C18E	4.3
C6A—C1A—C2A—C3A	−0.9 (6)	C17E—C16E—C15E—C14E	177.7
C6A—C7A—C8A—C9A	−7.3 (7)	C17E—C16E—C15E—C20E	−2.2
C6A—C7A—C8A—C13A	172.0 (4)	C17E—C18E—C19E—C20E	1.3
C6A—C7A—C20A—C15A	−170.4 (4)	C18E—C19E—C20E—C7E	179.6
C6A—C7A—C20A—C19A	9.0 (6)	C18E—C19E—C20E—C15E	0.7
C6B—C1B—C2B—C3B	4.8 (7)	C20E—C7E—C8E—C9E	−175.8
C6B—C7B—C8B—C9B	−2.8 (7)	C20E—C7E—C8E—C13E	5.5
C6B—C7B—C8B—C13B	178.2 (4)	C21A—O6A—C24A—C23A	11.8 (14)
C6B—C7B—C20B—C15B	−178.7 (4)	C21A—C22A—C23A—C24A	21.6 (14)
C6B—C7B—C20B—C19B	0.1 (7)	C22A—C23A—C24A—O6A	−21.8 (14)
C6C—C1C—C2C—C3C	0.6 (7)	C24A—O6A—C21A—C22A	2.1 (14)
C6C—C7C—C8C—C9C	2.4 (7)	C29A—C30A—C31A—C32A	175.6
C6C—C7C—C8C—C13C	−179.5 (4)	C29A—C30A—C35A—C34A	−175.5
C6C—C7C—C20C—C15C	178.7 (4)	C30A—C31A—C32A—C33A	0.1
C6C—C7C—C20C—C19C	−1.7 (6)	C31A—C30A—C35A—C34A	0.2
C6D—C1D—C2D—C3D	−0.1 (7)	C31A—C32A—C33A—C34A	0.1
C6D—C7D—C8D—C9D	−0.2 (7)	C32A—C33A—C34A—C35A	−0.1
C6D—C7D—C8D—C13D	179.8 (4)	C33A—C34A—C35A—C30A	−0.1
C6D—C7D—C20D—C15D	176.9 (4)	C35A—C30A—C31A—C32A	−0.2
C6D—C7D—C20D—C19D	−4.1 (7)	O6B—C21B—C22B—C23B	−21.5 (17)
C7A—C8A—C9A—C10A	179.4 (5)	C5F—C6F—C1F—O7	−176.7 (7)
C7A—C8A—C13A—C12A	179.7 (5)	C5F—C6F—C1F—C2F	0.0
C7A—C8A—C13A—C14A	0.4 (7)	C5F—C6F—C7F—C8F	102.9 (5)
C7B—C8B—C9B—C10B	−178.3 (5)	C5F—C6F—C7F—C20F	−73.1 (6)
C7B—C8B—C13B—C12B	178.7 (5)	C6F—C5F—C4F—C3F	0.0
C7B—C8B—C13B—C14B	0.1 (7)	C6F—C1F—C2F—C3F	0.0
C7C—C8C—C9C—C10C	177.5 (5)	C6F—C7F—C8F—C9F	1.9 (5)
C7C—C8C—C13C—C12C	−178.1 (5)	C6F—C7F—C8F—C13F	−179.2 (5)
C7C—C8C—C13C—C14C	0.0 (7)	C6F—C7F—C20F—C15F	−178.6 (5)
C7D—C8D—C9D—C10D	179.1 (5)	C6F—C7F—C20F—C19F	0.2 (5)
C7D—C8D—C13D—C12D	−179.0 (5)	C1F—C6F—C7F—C8F	−73.9 (6)
C7D—C8D—C13D—C14D	3.0 (7)	C1F—C6F—C7F—C20F	110.1 (5)
C8A—C7A—C20A—C15A	4.1 (6)	C1F—C2F—C3F—C4F	0.0
C8A—C7A—C20A—C19A	−176.5 (4)	C2F—C3F—C4F—C5F	0.0
C8A—C9A—C10A—C11A	1.5 (8)	C4F—C5F—C6F—C1F	0.0
C8A—C13A—C14A—C15A	0.1 (7)	C4F—C5F—C6F—C7F	−176.9 (7)
C8B—C7B—C20B—C15B	−0.8 (7)	C7F—C6F—C1F—O7	0.1 (6)
C8B—C7B—C20B—C19B	178.1 (4)	C7F—C6F—C1F—C2F	176.7 (7)
C8B—C9B—C10B—C11B	−0.7 (8)	C7F—C8F—C9F—C10F	179.6
C8B—C13B—C14B—C15B	−0.1 (7)	C7F—C8F—C13F—C12F	−179.2
C8C—C7C—C20C—C15C	−2.3 (6)	C7F—C8F—C13F—C14F	0.8
C8C—C7C—C20C—C19C	177.4 (4)	C8F—C7F—C20F—C15F	5.4
C8C—C9C—C10C—C11C	0.7 (10)	C8F—C7F—C20F—C19F	−175.8
C8C—C13C—C14C—C15C	−0.7 (8)	C8F—C9F—C10F—C11F	1.3
C8D—C7D—C20D—C15D	−2.5 (7)	C8F—C13F—C14F—C15F	−0.9
C8D—C7D—C20D—C19D	176.5 (4)	C9F—C8F—C13F—C12F	−0.3
C8D—C9D—C10D—C11D	−0.8 (9)	C9F—C8F—C13F—C14F	179.8
C8D—C13D—C14D—C15D	−1.8 (8)	C9F—C10F—C11F—C12F	−3.8
C9A—C8A—C13A—C12A	−1.0 (7)	C10F—C11F—C12F—C13F	4.3
C9A—C8A—C13A—C14A	179.7 (4)	C11F—C12F—C13F—C8F	−2.2
C9A—C10A—C11A—C12A	−2.1 (9)	C11F—C12F—C13F—C14F	177.7
C9B—C8B—C13B—C12B	−0.4 (7)	C12F—C13F—C14F—C15F	179.2
C9B—C8B—C13B—C14B	−179.0 (5)	C13F—C8F—C9F—C10F	0.7
C9B—C10B—C11B—C12B	0.2 (9)	C13F—C14F—C15F—C16F	−177.7
C9C—C8C—C13C—C12C	0.2 (7)	C13F—C14F—C15F—C20F	3.2
C9C—C8C—C13C—C14C	178.2 (5)	C14F—C15F—C16F—C17F	−178.3
C9C—C10C—C11C—C12C	−0.1 (11)	C14F—C15F—C20F—C7F	−5.5
C9D—C8D—C13D—C12D	1.0 (7)	C14F—C15F—C20F—C19F	175.7
C9D—C8D—C13D—C14D	−177.0 (5)	C15F—C16F—C17F—C18F	1.9
C9D—C10D—C11D—C12D	2.6 (10)	C16F—C15F—C20F—C7F	175.4
C10A—C11A—C12A—C13A	1.1 (10)	C16F—C15F—C20F—C19F	−3.4
C10B—C11B—C12B—C13B	0.2 (9)	C16F—C17F—C18F—C19F	−2.0
C10C—C11C—C12C—C13C	−0.5 (11)	C17F—C18F—C19F—C20F	−0.6
C10D—C11D—C12D—C13D	−2.6 (10)	C18F—C19F—C20F—C7F	−175.5
C11A—C12A—C13A—C8A	0.5 (9)	C18F—C19F—C20F—C15F	3.3
C11A—C12A—C13A—C14A	179.7 (6)	C20F—C7F—C8F—C9F	178.0
C11B—C12B—C13B—C8B	−0.1 (8)	C20F—C7F—C8F—C13F	−3.1
C11B—C12B—C13B—C14B	178.5 (5)	C20F—C15F—C16F—C17F	0.8
C11C—C12C—C13C—C8C	0.4 (9)	C21B—O6B—C24B—C23B	−32.3 (14)
C11C—C12C—C13C—C14C	−177.6 (6)	C21B—C22B—C23B—C24B	2.5 (16)
C11D—C12D—C13D—C8D	0.7 (8)	C22B—C23B—C24B—O6B	17.1 (14)
C11D—C12D—C13D—C14D	178.6 (6)	C24B—O6B—C21B—C22B	32.6 (16)
C12A—C13A—C14A—C15A	−179.2 (5)	C29B—C30B—C31B—C32B	175.6
C12B—C13B—C14B—C15B	−178.6 (5)	C29B—C30B—C35B—C34B	−175.4
C12C—C13C—C14C—C15C	177.3 (5)	C30B—C31B—C32B—C33B	0.0
C12D—C13D—C14D—C15D	−179.8 (5)	C31B—C30B—C35B—C34B	0.3
C13A—C8A—C9A—C10A	0.1 (7)	C31B—C32B—C33B—C34B	0.0
C13A—C14A—C15A—C16A	−177.4 (4)	C32B—C33B—C34B—C35B	0.1
C13A—C14A—C15A—C20A	1.5 (7)	C33B—C34B—C35B—C30B	−0.2
C13B—C8B—C9B—C10B	0.8 (8)	C35B—C30B—C31B—C32B	−0.2
C13B—C14B—C15B—C16B	−180.0 (5)	C43B—C44B—C45B—C46B	175.6
C13B—C14B—C15B—C20B	−0.4 (7)	C43B—C44B—C49B—C48B	−175.4
C13C—C8C—C9C—C10C	−0.8 (8)	C44B—C45B—C46B—C47B	0.0
C13C—C14C—C15C—C16C	−177.7 (5)	C45B—C44B—C49B—C48B	0.2
C13C—C14C—C15C—C20C	−0.1 (7)	C45B—C46B—C47B—C48B	0.0
C13D—C8D—C9D—C10D	−1.0 (8)	C46B—C47B—C48B—C49B	0.1
C13D—C14D—C15D—C16D	179.4 (5)	C47B—C48B—C49B—C44B	−0.2
C13D—C14D—C15D—C20D	−1.4 (7)	C49B—C44B—C45B—C46B	−0.1
C14A—C15A—C16A—C17A	−178.8 (5)	C36B—C37B—C38B—C39B	175.6
C14A—C15A—C20A—C7A	−3.6 (6)	C36B—C37B—C42B—C41B	−175.5
C14A—C15A—C20A—C19A	177.0 (4)	C37B—C38B—C39B—C40B	0.0
C14B—C15B—C16B—C17B	179.7 (5)	C38B—C37B—C42B—C41B	0.3
C14B—C15B—C20B—C7B	0.8 (7)	C38B—C39B—C40B—C41B	0.0
C14B—C15B—C20B—C19B	−178.1 (4)	C39B—C40B—C41B—C42B	0.0
C14C—C15C—C16C—C17C	176.5 (5)	C40B—C41B—C42B—C37B	−0.2
C14C—C15C—C20C—C7C	1.6 (7)	C42B—C37B—C38B—C39B	−0.2
C14C—C15C—C20C—C19C	−178.1 (4)	C36A—C37A—C38A—C39A	175.6
C14D—C15D—C16D—C17D	177.7 (5)	C36A—C37A—C42A—C41A	−175.5
C14D—C15D—C20D—C7D	3.6 (7)	C37A—C38A—C39A—C40A	0.0
C14D—C15D—C20D—C19D	−175.5 (4)	C38A—C37A—C42A—C41A	0.2
C15A—C16A—C17A—C18A	0.9 (7)	C38A—C39A—C40A—C41A	0.1
C15B—C16B—C17B—C18B	−1.6 (8)	C39A—C40A—C41A—C42A	0.0
C15C—C16C—C17C—C18C	1.0 (8)	C40A—C41A—C42A—C37A	−0.1
C15D—C16D—C17D—C18D	−1.9 (8)	C42A—C37A—C38A—C39A	−0.2
C16A—C15A—C20A—C7A	175.3 (4)		
