# Supplementary material for: Crystal structure of a binuclear mixed-valence ytterbium complex containing a 2-anthracene-substituted phenoxide ligand
Source: Acta Crystallogr E Crystallogr Commun. 2019 Aug 23;75(Pt 9):1367–71. doi: 10.1107/S205698901901154X (PMC6727046; doi:10.1107/S205698901901154X)
Supplement: Supplementary file 3 [file e-75-01367-sup3.pdf]

STRUCTURE REPORTS

Volume 75 (2019)

Supporting information for article:

Crystal structure of a binuclear mixed-valence Ytterbium complex containing a 2-anthracene-substituted phenoxide ligand

David J. Berg and Brendan Twamley

Crystal Structure of a Dinuclear, Mixed Valence Ytterbium Complex Containing an 2-Anthracene-substituted Phenoxide Ligand.

David J. Berg and Brendan Twamley

CSD reference codes and literature citations for structures containing non-chelating aryloxides bound to 6-coordinate Yb^{3+} for comparison of Yb-O(aryloxide) distances:

Bridging aryloxide:

FAPPES Deacon, G. B., Forsyth, C. M., Harika, R., Junk, P. C., Ziller, J. W. & Evans, W. J. (2004) J. Mater. Chem. **14**, 3144
OFOQOP Carretas, J., Branco, J., Marcalo, J., Isolani, P., Domingos, A. & de Matos, A. P. (2001) J. Alloys Compd. **323**, 169

Terminal aryloxide:

BEVWIK Tu, J., Li, W., Xue, M., Zhang, Y. & Shen, Q. (2013) Dalton Trans. **42**, 5890
BEVWOK Tu, J., Li, W., Xue, M., Zhang, Y. & Shen, Q. (2013) Dalton Trans. **42**, 5890
BEVWUW Tu, J., Li, W., Xue, M., Zhang, Y. & Shen, Q. (2013) Dalton Trans. **42**, 5890
COGLAL Yao, Y., Shen, Q. & Sun, J. (1998) Polyhedron **17**, 519
CONBEN Deacon, G. B., Junk, P. C. & Moxey, G. J. (2008) Z. Anorg. Allg. Chem. **634**, 2789
DASPEU Hamidi, S., Deacon, G. B., Junk, P. C. & Neumann, P. (2012) Dalton Trans. **41**, 3541
GAKJOT Nie, K., Gu, X., Yao, Y., Zhang, Y. & Shen, Q. (2010) Dalton Trans. **39**, 6832
HAWCEP Chen, P., Li, Q., Chen, S., Yan, P., Wang, Y. & Li, G. (2012) Inorg. Chem. Commun. **17**, 17
JEMRUO Deacon, G. B., Nickel, S., MacKinnon, P. & Tiekink, E. R. T. (1990) Aust. J. Chem. **43**, 1245
NOBRED Yang, S., Nie, K., Zhang, Y., Xue, M., Yao, Y. & Shen (2014) Inorg. Chem. **53**, 105
NOFVEJ Yao, Y., Shen, Q., Sun, J. & Xue, F. (1998) Acta Cryst., Sect. C: Cryst. Struct. Commun. **54**, 625
NUZGEV Chen, H., Liu, P., Yao, H., Zhang, Y., Yao, Y. & Shen, Q. (2010) Dalton Trans. **39**, 6877
NUZGIV Chen, H., Liu, P., Yao, H., Zhang, Y., Yao, Y. & Shen, Q. (2010) Dalton Trans. **39**, 6877
PASQAE Chen, Z., Gu, W., Wang, Y. & Yao, Y. (2017) Polyhedron **134**, 22
QUCYUI Deacon, G. B., Forsyth, C.M. & Wilkinson, D. L. (2001) Chem. Eur. J. **7**, 1784
RIJRUX Deacon, G. B., Feng, T., Junk, P. C., Skelton, B. W. & White, A. W. (1997) Chem. Ber. **130**, 851
SUKHEM Deacon, G. B., Junk, P. C. & Moxey, G. J. (2009) Chem. Asian J. **4**, 1717
XASPEO Rabe, G. W., Riederer, F. A. & Yap, G. P. A. (2010) CSD-Communications: private communication
XAZKAK Deacon, G. B., Forsyth, C. M. &Scott, N. M. (2000) Eur. J. Inorg. Chem. pp. 2501
YIHCEP Fan, L., Wang, Y., Yao, Y., Wu, B. & Shen, Q. (2013) Z. Anorg. Allg. Chem. **639**, 739
YUBBON Norton, K., Kumar, G. A., Emge, T. J., Riman, R. E., Brik, M. G. & Brennan, J. G. (2009) Inorg. Chem. **48**, 3573

CSD reference codes and literature citations for structures containing non-chelating aryloxides bound to 5-coordinate Yb^{2+} for comparison of Yb-O(aryloxide) distances:

Bridging aryloxide:

HOCFUA Deacon, G. B., Forsyth, C. M., Junk, P. C., Skelton, B. W. & White, A. H. (1999) Chem. Eur. J. **5**, 1452
ZOQFAO Li, J., Hao, J. & Cui, C. (2015) Dalton Trans. **44**, 767

Terminal aryloxide:

ESUJAD Morissette, M., Haule, S., McDonald, R., Ferrence, G. M. & Takats, J. (2004) Polyhedron **23**, 263
ESUJEH Morissette, M., Haule, S., McDonald, R., Ferrence, G. M. & Takats, J. (2004) Polyhedron **23**, 263
FIDQEO Trifonov, A. A., Kirilov, E. N., Fedorova, E. A., Makarenko, N. P., Bochkarev, M. N., Shumann, H. & Muehle, S. (1998) Russ. Chem. Bull. pp. 2345
IBIFAA Deacon, G. B., Fanwick, P. E., Gitlits, A., Rothwell, I. P., Skelton, B. W. & White, A. H. (2001) Eur. J. Inorg. Chem. pp. 1505
KEDRUH Deacon, G. B., Fallon, G. D., Forsyth, C. M., Harris, S. C., Junk, P. C., Skelton, B. W. & White, A. H. (2006) Dalton Trans. p. 802.
PEFMUI Deacon, G. B., Feng, T., MacKinnon, P., Newnham, R. H., Nickel, S., Shelton, B. W. & White, A. H. (1993) Aust. J. Chem. **46**, 387
RIJROR Deacon, G. B., Feng, T., Junk, P. C., Skelton, B. W. & White, A. W. (1997) Chem. Ber. **130**, 851
VATTAL Deacon, G. B., Hitchcock, P. B., Holmes, S. A., Lappert, M. F., MacKinnon, P. & Newnham, R. H. (1989) Chem. Commun. pp. 935

CSD reference codes and literature citations for structures containing THF bound to 6-coordinate Yb^{3+} for comparison of Yb-O(THF) distances:

BENXOI Yu, L., Yao, Y., Shen, Q., Zhang, J., Wu, L. & Ye, L. (2003) Chin. J. Chem. **21**, 442.
BINVOK Li, H., Xu, F., Chen, J., Cheng, M., Zhang, Y., Zhang, W., Lang, J. & Shen, Q. (2004) J. Organomet. Chem. **689**, 3438.
BUJCEP Cheng, Y., Hitchcock, P. B., Khvostov, A. V. & Lappert, M. F. (2009) Inorg. Chim. Acta **362**, 4678.
BUJXAH Gu, W., Xu, P., Wang, Y., Yao, Y., Yuan, D. & Shen, Q. (2015) Organometallics **34**, 2907.
BUJXIP Gu, W., Xu, P., Wang, Y., Yao, Y., Yuan, D. & Shen, Q. (2015) Organometallics **34**, 2907.
BUQXIV Yao, S.., Chan, H., Lam, C. & Lee, H. (2009) Inorg. Chem. **48**, 9936.
BUQXUH Yao, S.., Chan, H., Lam, C. & Lee, H. (2009) Inorg. Chem. **48**, 9936.
CESHAL Li, W., Zhang, Z., Yao, Y., Zhang, Y. & Shen, Q. (2012) Organometallics **31**, 3499.
CIFKEK de Bruin-Dickason, C. N., Boutland, A. J., Dange, D., Deacon, G. B. & Jones, C. (2018) Dalton Trans. **47**, 9512.
CIFXUL Meyer, N. & Roesky, P. W. (2007) Dalton Trans. p. 2652.
CIKVOI Meyer, N. & Roesky, P. W. (2007) Dalton Trans. p. 2652.
COGLAL Yao, Y., Shen, Q. & Sun, J. (1998) Polyhedron **17**, 519.
COHNIY Wang, Z., Bian, Q., Liu, S., Liu, T., Huang, H., Yanjiu, H. & Yu, Y. (2012) Chem. Res. Appln. (Chin) **24**, 1571.
CUBNEU Deacon, G. B., Junk, P. C., Wang, J. & Werner, D. (2014) Inorg. Chem. **53**, 12553.
DEHVES Zhou, L., Yao, Y., Li, C., Zhang, Y. & Shen, Q. (2006) Organometallics **25**, 2880.
DUKQEG Emge, T. J., Kornienko, A. & Brennan, J. G. (2009) Acta Crystallogr., Sect. C: Cryst. Struct. Commun. **65**, m422.
EFENIN Cheng, J. Saliu, K., Kiel, G.Y., Ferguson, M. J., McDonald, R. & Takats, J. (2008) Angew. Chem., Int. Ed. **47**, 4910.
EFENIN01 Rabe, G., Riederer, F. A. & Yap, G. P. A. (2010) CSD Communication (Private Communication).
EGOCAE Deacon, G. B., Evans, D. J. & Junk P. C. (2002) Z. Anorg. Allg. Chem. **628**, 2033.
EREVIH Richtera, L., Jancik, V., Hermanova, S., Krpoun, K. & Thompson-Montero, K. (2011) Acta Crystallogr., Sect.E: Struct. Rep. Online **67**, m700.
EVEQAX Xue, M., Yao, Y., Zhang, Y., Shen, Q. & Huaxue, J. (2004) Chin. J. Struct. Chem. (Chin) **23**, 275.
FAFXAM Niemeyer, M. (2002) Z. Anorg. Allg. Chem. **628**, 647.
FAPPES Deacon, G. B., Forsyth, C. M., Harika, R. Junk, P. C., Ziller, J. W. & Evans, W. H. (2004) J. Mater. Chem. **14**, 3144.
FIQYEK Xu, X., Ma, M., Yao, Y., Zhang, Y. & Shen, Q. (2005) Eur. J. Inorg. Chem. p. 676.
FUHVIP Zhao, L., Ding, H., Zhao, B., Lu, C. & Yao, Y. (2014) Polyhedron **83**, 50.
GAKJOT Nie, K., Gu, X., Yao, Y., Zhang, Y. & Shen, Q. (2010) Dalton Trans. **39**, 6832.
GIJXED Zhou, L., Wang, J., Zhang, Y., Yao, Y. & Shen, Q. (2007) Inorg. Chem. **46**, 5763.
HOGDEO Qin, J., Wang, P., Li, Q., Zhang, Y., Yuan, D. & Yao, Y. (2014) Chem. Commun. **50**, 10952.
HOPWAL Xu, H., Qi, R., Xu, B., Yao, Y., Nie, K., Zhang, Y. & Shen, Q. (2009) Polyhedron, **28**, 574.
HOZMIT Zhang, Z., Xu, X., Li, W., Yao, Y., Yong, Z., Shen, Q. & Luo, Y. (2009) Inorg. Chem. **48**, 5715.
IKUWAN Panda, T. K., Kaneko, H., Pal, K., Tsurugi, H. & Mashima, K. (2010) Organometallics **29**, 2610.
JOKCOC Qi, R., Liu, B., Xu, X., Yang, Z., Yao, Y., Zhang, Y. & Shen, Q. (2008) Dalton Trans. p. 5016.
JOKCUI Qi, R., Liu, B., Xu, X., Yang, Z., Yao, Y., Zhang, Y. & Shen, Q. (2008) Dalton Trans. p. 5016.
JOKDET Qi, R., Liu, B., Xu, X., Yang, Z., Yao, Y., Zhang, Y. & Shen, Q. (2008) Dalton Trans. p. 5016.
KAGWOF Yao, Y., Xue, M., Luo, Y., Zhang, Z., Jiao, R., Zhang, Y., Shen, Q., Wong, W., Yu, K. & Sun, J. (2003) J. Organomet. Chem. **678**, 108.
KANSUQ Marshall, G., Wooles, A. J., Mills, D. P., Lewis, W., Blake, A. J. & Liddle, S. T. (2013) Inorganics **1**, 46.
KIRFEY Shestakov, B. G., Makhrova, T. V., Lyssenko, K. A. & Trifonov, A. A. (2013) Russ. Chem. Bull. (Russ) p. 414.
KUGKII Wang, S., Zhang, G., Zhou, S. & Wei, Y. CSD Communication (Private Communication) 2015.
LASGAO Yao, Y., Ma, M., Xu, X., Zhang, Y., Shen, Q. & Wong, W. (2005) Organometallics **24**, 4014.
LASGIW Yao, Y., Ma, M., Xu, X., Zhang, Y., Shen, Q. & Wong, W. (2005) Organometallics **24**, 4014.
LASGOC Yao, Y., Ma, M., Xu, X., Zhang, Y., Shen, Q. & Wong, W. (2005) Organometallics **24**, 4014.
MAJMAL Apostolidis, C., Carvalho, A., Domingos, A., Kanellakopulos, B., Maier, R., Marques, N., Pires de Matos, A. & Rebizant, J. (1999) Polyhedron 18, 263.
MIQJED Yuan, F., Zhou, Y, Li, L. & Zhu, X. (2013) Inorg. Chim. Acta **408**, 33.
NELMUM Niemeyer, M. (2001) Acta Crystallogr., Sect. E: Struct. Rep. Online **57**, m363.
NIRTIS Xu, X., Zhang, Z., Yao, Y., Zhang, Y. & Shen, Q. (2007) Inorg. Chem. **46**, 9379.
NIVFAA Wang, Q., Xiang, L. & Zi, G. (2008) J. Organomet. Chem. **693**, 68.
NOBRED Yang, S., Nie, K., Zhang, Y., Xue, M., Yao, Y. & Shen, Q. (2014) Inorg. Chem. **53**, 105.
NOSJOU Bochkarev, L. N., Zheleznova, T. A., Safronova, A. V., Drozdov, M. S., Zhil'tsov, S. F., Zakharov, L. N., Fukin, G. K. & Khorshev, S. Y. (1998) Russ. Chem. Bull. (Russ) p. 163.
NOSJOU01 Deacon, G. B. & Forsyth, C. M. (2003) Organometallics **22**, 1349.
NUZGEV Chen, H., Liu, P., Yao, H., Zhang, Y., Yao, Y. & Shen, Q. (2010) Dalton Trans. **39**, 6877.
NUZGIZ Chen, H., Liu, P., Yao, H., Zhang, Y., Yao, Y. & Shen, Q. (2010) Dalton Trans. **39**, 6877.
OBITID Zhang, Z., Yao, Y., Zhang, Y., Shen, Q. & Wong, W. (2004) Inorg. Chim. Acta **357**, 3173.
OCUGIE Xu, B., Huang, L., Yang, Z., Yao, Y., Zhang, Y. & Shen, Q. (2011) Organometallics **30**, 3588.
ODIFAI Yao, Y., Zhang, Y., Shen, Q. & Yu, K. (2002) Organometallics **21**, 819.
OFOQOP Carretas, J., Branco, J., Marcalo, J., Isolani, P., Domingos, A. & .de Matos, A. P. (2001) J. Alloys Compd. **323**, 169.
OLALIX Luo, Y., Li, W., Lin, D., Yao, Y., Zhang, Y. & Shen, Q. (2010) Organometallics **29**, 3507.
PAVGUR Nie, K., Wang, C., Han, Y., Zhang, J. & Yao, Y. (2017) Inorg. Chim. Acta **466**, 228.
PEDJIR Qian, C., Wang B., Deng, D., Xu, C., Sun, X., Ling, R. & Jiegou H. (1993) Chin. J. Struct. Chem. (Chin) **12**, 18.

PEDJIR02 Deacon, G. B., Feng, T., Junk, P. C., Skelton, B. W., Sobolev, A. N. & White, A. H. (1998) Aust. J. Chem. **51**, 75.
PELKAV Ma, Y., Bestgen, S., Gamer, M. T., Konchenko, S. N. & Roesky, P. W. (2017) Angew. Chem., Int. Ed. **56**, 13249.
PETYES Deacon, G. B., Feng, T., Nickel, S., Skelton, B. W. & White, A. H. (1993) Chem. Commun. p. 1328.
PUQPUN Wooles, A. J., Mills, D. P., Lewis, W., Blake, A. J. & Liddle, S. T. (2010) Dalton Trans. **39**, 500.
QIGJIC Ma, Y., Pushkarevsky, N., Sukhikh, T. S., Galashov, A. E., Makarov, A. G., Roesky, P. W. & Konchenko, S. N. (2018) Eur. J. Inorg. Chem. p. 3388.
QIGJUO Ma, Y., Pushkarevsky, N., Sukhikh, T. S., Galashov, A. E., Makarov, A. G., Roesky, P. W. & Konchenko, S. N. (2018) Eur. J. Inorg. Chem. p. 3388.
QIGKEZ Ma, Y., Pushkarevsky, N., Sukhikh, T. S., Galashov, A. E., Makarov, A. G., Roesky, P. W. & Konchenko, S. N. (2018) Eur. J. Inorg. Chem. p. 3388.
QIHRIK Xu, B., Han, X., Yao, Y. Zhang, Y. & Shen, Q. (2010) Chin. J. Chem. **28**, 1013.
QUCYUI Deacon, G. B., Forsyth, C. M. & Wilkinson, D. L. (2001) Chem.-Eur. J. **7**, 1784.
QUCZUJ Deacon, G. B., Forsyth, C. M. & Wilkinson, D. L. (2001) Chem.-Eur. J. **7**, 1784.
RACFAD Burgstein, M. R. & Roesky, P. W. (2003) Organometallics **22**, 1372.
RECZOQ Benndorf, P., Kratsch, J., Hartenstein, L., Preuss, C. M. & Roesky, P. W. (2012) Chem.-Eur. J. **18**, 14454.
REGZUA Zhu, X., Zhou, S., Wang, S., Wei, Y., Zhang, L., Wang, F., Wang, S. & Feng, Z. (2012) Chem. Commun. **48**, 12020.
RIGTOQ Deacon, G. B., Feng, T., Junk, P. C., Meyer, G., Scott, N. M., Skelton, B. W. & White (2000) Aust. J. Chem. **53**, 853.
RIKSOT Anfang, S., Karl, M., Faza, N., Massa, W., Magull, J. & Dehnicke, K. (1997) Z. Anorg. Allg. Chem. **623**, 1425.
RIKYAN Kratsch, J., Kuzdrowska, M., Schmid, M., Kazeminejad, N., Kaub, C., Ona-Burgos, P., Guillaume, S. M. & Roesky, P. W. (2013) Organometallics 32, 1230.
SUKHEM Deacon, G. B., Junk, P. C. & Moxey, G. J. (2009) Chem. Asian J. **4**, 1717.
TOKWAS Giessmann, S., Blaurock, S., Edelmann, A., Lorenz, V. & Edelmann, F. T. (2008) Z. Anorg. Allg. Chem. **634**, 2459.
UBUGAZ Niemeyer, M. (2001) Eur. J. Inorg. Chem. p. 1969.
UROKOC Huang, L., Han, X., Yao, Y., Zhang, Y. & Shen, Q. (2011) Appl. Organomet. Chem. **25**, 464.
UWAXIB Zou, J., Berg, D. J., Stuart, D., McDonald, R., Twamley, B. (2011) Organometallics **30**, 4958.
VETYUO Wedler, M., Noltemeyer, M., Pieper, U., Schmidt, H., Stalke, D. & Edelmann, F. T. (1990) Angew. Chem., Int. Ed. **29**, 894.
VOSKAP Wedler, M., Recknagel, A., Gilje, J. W., Noltemeyer, M. & Edelmann, F. T. (1992) J. Organomet. Chem. **426**, 295.
WAHHUK Krogh-Jespersen, K., Romanelli, M. D., Melman, J. H., Emge, T. J. & Brennan, J. G. (2010) Inorg. Chem. **49**, 552.
WAQXAO Yao, Y., Xu, X., Liu, B., Zhang, Y., Shen, Q. & Wong, W. (2005) Inorg. Chem. **44**, 5133.
WEKRUB Ku, K., Au, C., Chan, H. & Lee, H. (2013) Dalton Trans. **42**, 2841.
WEWCUY Gu, X., Han, X., Yao, Y. Zhang, Y. & Shen, Q. (2010) J. Organomet. Chem. **695**, 2726.
XASPEO Rabe, G. W., Riederer, F. A. & Yap, G. P. A. (2010) CSD Communication (Private Communication).
XAZKAK Deacon, G. B., Forsyth, C. M. & Scott, N. M. (2000) Eur. J. Inorg. Chem. p. 2501.
XETLIT Cole, M. L., Deacon, G. B., Forsyth, C. M., Junk, P. C., Konstas, K., Wang, J., Bittig, H. & Werner, D. (2013) Chem.-Eur. J. **19**, 1410.
XETMEQ Cole, M. L., Deacon, G. B., Forsyth, C. M., Junk, P. C., Konstas, K., Wang, J., Bittig, H. & Werner, D. (2013) Chem.-Eur. J. **19**, 1410.
XETNER Cole, M. L., Deacon, G. B., Forsyth, C. M., Junk, P. C., Konstas, K., Wang, J., Bittig, H. & Werner, D. (2013) Chem.-Eur. J. **19**, 1410.
YIHSEP Fan, L., Wang, Y., Yao, Y., Wu, B. & Shen, Q. (2013) Z. Anorg. Allg. Chem. **639**, 739.
YIYHUJ Evans, W. J., Shreeve, J. L., Ziller, J. W. & Doedens, R. J. (1995) Inorg. Chem. **34**, 576.
YUBBON Norton, K., Kumar, G. A., Dilks, J. L., Emge, T. J., Riman, R. E., Brik, M. G. & Brennan, J. G. (2009) Inorg. Chem. **48**, 3573.
YUHJES Guo, L., Zhu, X., Zhang, G., Wei, Y., Zhou, S., Feng, Z. & Wang, S. (2015) Inorg. Chem. **54**, 5725.
YUKWIM Cheng, H., Xiao, Y., Lu, C., Zhao, B., Wang, Y. & Yao, Y. (2015) New J. Chem. **39**, 7667.
ZACQAV Evans, W. J., Ansari, M. A. & Ziller, J. W. (1995) Inorg. Chem. **34**, 3079.
ZAPGUV Werner, D., Zhao, X., Best, S. P., Maron, L., Junk, P. C., Deacon, G. B. (2017) Chem.-Eur. J. **23**, 2084.
ZAPHIK Werner, D., Zhao, X., Best, S. P., Maron, L., Junk, P. C., Deacon, G. B. (2017) Chem.-Eur. J. **23**, 2084.
ZAPHOQ Werner, D., Zhao, X., Best, S. P., Maron, L., Junk, P. C., Deacon, G. B. (2017) Chem.-Eur. J. **23**, 2084.
ZAPJAE Werner, D., Zhao, X., Best, S. P., Maron, L., Junk, P. C., Deacon, G. B. (2017) Chem.-Eur. J. **23**, 2084.
ZAPJOS Werner, D., Zhao, X., Best, S. P., Maron, L., Junk, P. C., Deacon, G. B. (2017) Chem.-Eur. J. **23**, 2084.
ZIXZIP Geissinger, M. & Magull, J. (1995) Z. Anorg. Allg. Chem. **621**, 2043.

CSD reference codes and literature citations for structures containing arene ligands π-bonded to Yb^{2+} for comparison of Yb-C contact distances:

AFIKIJ Hou, Z., Koizumi, T., Nishiura, M. & Wakatsuki, Y. (2001) Organometallics **20**, 3323.
CIFJOT de Bruin-Dickason, C. N., Boutland, A. J., Dange, D., Deacon, G. B. & Jones, C. (2018) Dalton Trans. **47**, 9512.
CIFJUZ de Bruin-Dickason, C. N., Boutland, A. J., Dange, D., Deacon, G. B. & Jones, C. (2018) Dalton Trans. **47**, 9512.
CUDNEV01 Yan, K. Schoendorff, G., Upton, B. M., Ellern, A., Windus, T. L. & Sadow, A. D. (2013) Organometallics **32**, 1300.
FUPZEW Deacon, G. B., Junk, P. C., Moxey, G. J., Ruhlandt-Senge, K., St.Prix, C. & Zuniga, M. F. (2009) Chem.-Eur. J. **15**, 5503.
GIRJUP Schuhknecht, D., Truong, K., Spaniol, T. P., Maron, L. & Okuda, J. (2018) Chem. Commun. **54**, 11280.
HOCFUA Deacon, G. B., Forsyth, C. M., Junk, P. B., Skelton, B. W. & White, A. H. (1999) Chem.-Eur. J. **5**, 1452.
HOCGAH Deacon, G. B., Forsyth, C. M., Junk, P. B., Skelton, B. W. & White, A. H. (1999) Chem.-Eur. J. **5**, 1452.
ILEVAW Muller-Buschbaum, K. & Quitmann, C. C. (2003) Z. Anorg. Allg. Chem. **629**, 1610.
LIBGIN Evans, W. J., Champagne, T. M. & Ziller, J. W. (2007) Organometallics **26**, 1204.
OGONAZ Deacon, G. B. & Forsyth, C. M. (2002) Chem. Commun. p. 2522.
OGONED Deacon, G. B. & Forsyth, C. M. (2002) Chem. Commun. p. 2522.
PETJUU Heitmann, D., Jones, C., Junk, P. C., Lippert, K. & Stasch, A. (2007) Dalton Trans. p. 187.
RIKJIG Basalov, I. V., Lyubov, D. M., Fukin, G. K., Cherkasov, A. V. & Trifonov, A. A. (2013) Organometallics **32**, 1507.
RIKKON Basalov, I. V., Lyubov, D. M., Fukin, G. K., Cherkasov, A. V. & Trifonov, A. A. (2013) Organometallics **32**, 1507.
TAJSOP Basalov, I. V., Yurova, O. S., Cherkasov, A. V., Fukin, G. K. & Trifonov, A. A. (2016) Inorg. Chem. **55**, 1236.
TAXWIZ Hauber, S. & Niemeyer, M. (2005) Inorg. Chem. **44**, 8644.
UBUFUS Niemeyer, M. (2001) Eur. J. Inorg. Chem. p. 1969.
VIVSOK Tolpygin, A. O., Cherkasov, A. V., Fukin, G. K. & Trifonov, A. A. (2014) Inorg. Chem. **53**, 1537.
YAMGOJ Deacon, G. B., Forsyth, C. M. & Junk, P. C. (2005) Eur. J. Inorg. Chem. p. 817.
YAMGUP Deacon, G. B., Forsyth, C. M. & Junk, P. C. (2005) Eur. J. Inorg. Chem. p. 817.

Supplementary information file S2

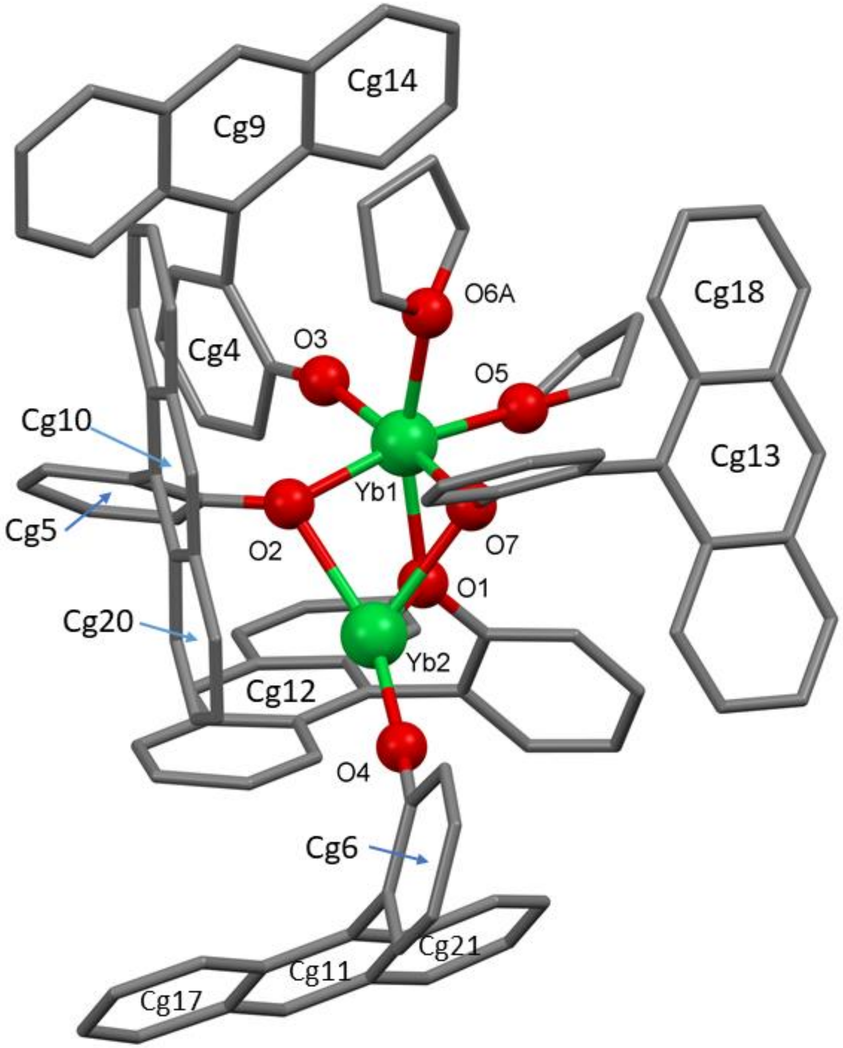

Table S2: C.H…π interactions

D—H···*A*	*D*—H	H···*A*	*D*···*A*	*D*—H···*A*
C2A—H2A···Cg12	0.95	2.99	3.832 (5)	148
C18D—H18D···Cg11	0.95	2.60	3.202 (5)	122
C19B—H19B···Cg5	0.95	3.00	3.680 (5)	130
C19D—H19D···Cg21	0.95	2.96	3.542 (5)	121
C21A—H21A···Cg14	0.99	2.73	3.642 (19)	154
C24A—H24B···Cg24	0.99	2.35	3.276 (11)	156
C28—H28B···Cg18	0.99	2.62	3.497 (5)	147
C2F—H2F···Cg10	0.95	2.70	3.490 (6)	141
C9F—H9F···Cg8	0.95	2.36	3.047 (5)	129
C21B—H21C···Cg14	0.99	2.52	3.43 (2)	153
C24B—H24C···Cg9	0.99	2.91	3.672 (12)	134
C10A—H10A···Cg4^{i}	0.95	2.68	3.520 (5)	148
C11E—H11E···Cg30^{i}	0.95	2.89	3.572 (6)	130
C14A—H14A···Cg21^{ii}	0.95	2.71	3.626 (5)	163

C16A—H16A···Cg11^{ii}	0.95	2.73	3.648 (5)	163
C17E—H17E···Cg17^{iii}	0.95	2.72	3.638 (4)	162
C23A—H23A···Cg27^{iv}	0.99	2.80	3.682 (12)	149
C26—H26B···Cg28^{v}	0.99	2.73	3.672 (7)	158
C26—H26B···Cg31^{v}	0.99	2.81	3.762 (9)	162
C29A—H29A···Cg13^{vi}	0.98	2.78	3.507 (10)	131
C29A—H29A···Cg25^{vi}	0.98	2.64	3.485 (10)	144
C32A—H32A···Cg12^{vii}	0.95	2.63	3.489 (5)	151
C36A—H36D···Cg6^{vii}	0.98	2.86	3.770 (8)	154
C16F—H16F···Cg20^{v}	0.98	2.86	3.734 (5)	153
Symmetry codes: (i) −*x*, −*y*+1, −*z*+1; (ii) *x*, −*y*+3/2, *z*+1/2; (iii) −*x*, *y*+1/2, −z+1/2; (iv) −*x*+1, −*y*+1, −*z*+1; (v) *x*−1, *y*, *z*; (vi) *x*+1, *y*−1, *z*; (vii) −*x*+1, *y*−1/2, −z+1/2.				

C4g is the centroid of ring C1A-C6A
C5g is the centroid of ring C1B-C6B
C6g is the centroid of ring C1C-C6C
C9g is the centroid of ring C7A/C8A/C13A-C15A/C20A
C10g is the centroid of ring C7B/C8B/C13B-C15B/C20B
C11g is the centroid of ring C7C/C8C/C13C-C15C/C20C
C12g is the centroid of ring C7D/C8D/C13D-C15D/C20D
C13g is the centroid of ring C7E/C8E/C13E-C15E/C20E

C14g is the centroid of ring C8A-C13A
C17g is the centroid of ring C8B-C13B
C18g is the centroid of ring C8E-C13E
C20g is the centroid of ring C15B-C20B
C21g is the centroid of ring C15C-C20C
C24g is the centroid of ring C1F-C6F (minor component of Cg6)
C25g is the centroid of ring C7F/C8F/C13F-C15F/C20F (minor component of Cg11)
C27g is the centroid of ring C30B-C35B (toluene solvate molecule)
C28g is the centroid of ring C37A-C42A (toluene solvate molecule)
C30g is the centroid of ring C44B-C49B (toluene solvate molecule)
C31g is the centroid of ring C37B-C42B (toluene solvate molecule)
